# Techniques to Improve the Performance of Planar Microwave Sensors: A Review and Recent Developments

**DOI:** 10.3390/s22186946

**Published:** 2022-09-14

**Authors:** Mohammad Abdolrazzaghi, Vahid Nayyeri, Ferran Martin

**Affiliations:** 1 Electrical and Computer Engineering Department, University of Toronto, 10 King’s College Circle, Toronto, ON M5S3G4, Canada; 2 School of Advanced Technologies, Iran University of Science and Technology, Tehran 1684613114, Iran; 3 Centro de Investigación en Metamateriales para la Innovación en Tecnologías Electrónica y de Comunicaciones (CIMITEC), Departament d’Enginyeria Electrònica, Universitat Autònoma de Barcelona, 08193 Bellaterra, Spain

**Keywords:** microwave sensor, dielectric characterization, microfluidics, sensitivity, resolution, active sensor, machine learning, resonators

## Abstract

Planar microwave sensors have become increasing developed in recent decades, especially in material characterization (solid/liquid) as they provide regions highly sensitive to the surrounding medium. However, when it comes to deciphering the content of practical biological analytes or chemical components inside a host medium, even higher sensitivities are required due to their minute concentrations. This review article presents a comprehensive outlook on various methodologies to enhance sensitivity (e.g., coupling resonators, channel embedding, analyte immobilization, resonator pattern recognition, use of phase variation, using coupled line section, and intermodulation products), resolution (active sensors, differential measurements), and robustness (using machine learning) of arbitrary sensors of interest. Some of the most practical approaches are presented with prototype examples, and the main applications of incorporating such procedures are reported. Sensors with which the proposed techniques are implemented exhibit higher performance for high-end and real-life use.

## 1. Introduction

Electromagnetic (EM) wave propagation in a medium depends on its electric and magnetic properties, which can be summarized as complex-valued permittivity and permeability [1]. The desired operation of a microwave device relies on the comprehensive understanding of the constituent substrate properties. Due to the propagation nature of these wavelengths, the surrounding medium also affects the optimal operation. Material properties determine the key role of many applications such as medicine, quality control (e.g., water), agriculture [2,3], smart home [4], etc. The microwave measurement of dielectric properties in a medium has been explored in recent decades as an alternative to the most established optical methods. With huge investments in the development of microwave sensing, alternative methodologies have been investigated to replace commonly used and expensive sensing apparatus including fiber optics, wearable sensors, image sensors, chemical and biosensors [5,6,7,8]. It is found that, compared to other methods, microwave devices bring about highly sensitive regions to the surrounding medium including solid, liquid, and even gaseous compositions [9,10]. The interaction of EM waves with material, even remotely, is intensified in the desired regions, which makes them useful for applications that require distant sensing [11,12,13]—specifically in applications involving noninvasive near-surface measurements such as soil moisture sensing using time-domain reflectometry [14,15]. Contact sensors reduce their lifespan which adds to their regular maintenance cost while being continuously used, or increases the supplement cost while carrying consumable and disposable parts [16,17]. Non-contact sensing as well as small size, compactness, low-cost design, and robust sensing significantly increase microwave sensor performance in complex permittivity measurements [18,19,20,21,22,23], while facilitating its integration into monolithic microwave integrated circuits (MMIC) [24,25,26]. These sensors take different topologies including split-ring resonator (SRR) [20] and complementary SRR (CSRR) [27,28]. They are further improved to cope with common sources of noise/error with differential sensing [29,30]. For some cases where the frequency of resonance shift is negligible, either the amplitude of resonance is measured instead [31] or the resonance profile sharpness is enhanced with compensating the power loss using electronics [32].

In this review paper, several techniques are discussed that can be applied to resonant-based sensors for material (solid/liquid/gaseous) composition characterization. Even though planar structures are more favorable in the practical prototypes presented here, mainly because of their low profile and versatility compared with the bulky cavity counterparts, the proposed procedures are considered general methods that can be applied to a broader range of sensors including non-planar and non-resonant ones. First, the sensitivity of sensors is discussed, which is of the utmost importance in biosensors and high-end applications and practical methods are presented to improve a sensor’s sensitivity including coupled resonators [33,34], the use of embedded channels inside substrates [35], analyte immobilization [36], resonator pattern optimization [37], using phase variation [38,39], coupled-line sections [40], and nonlinearity and intermodulation products [41]. Second, for a sensor with given sensitivity, the performance can be improved with respect to the output resolution while compensating the sensor loss by the active resonance method, using the sensor in oscillatory mode, or employing differential measurements in cross-mode [32,42,43,44,45,46]. Finally, the intrinsic feature of wave-propagation for planar designs makes them susceptible to environmental variations. Therefore, machine learning is proposed as a means to grasp the possible impacts and remove them from the sensor response towards a robust design [47,48,49,50].

## 2. Sensitivity Enhancement

### 2.1. Planar Structures in Frequency-Variation Sensors

In this section, it is shown that sensitivity in frequency-variation microwave sensors implemented in planar technology can be optimized by adequately choosing the resonant element. Such a resonant element can be either a metallic resonator or a slot (also called defect-ground-structure DGS) resonator (a review of different electrically small resonant elements for sensing can be found in [51]). Planar frequency-variation sensors can be implemented as transmission-mode or reflective-mode structures, but transmission-mode frequency-variation sensors are more common [52]. Moreover, although transmission-mode frequency-variation sensors can operate either as notch filters or as bandpass filters, the former dominate the available literature. In this section, this study refers to sensors operating as transmission-mode notch filters, the notch being caused by the coupling (electric, magnetic or mixed) between the resonant element and the host transmission line. The consideration of a metallic or a slot resonator for sensor implementation may depend on several criteria. Nevertheless, slot resonators etched in the ground plane of a microstrip line exhibit an inherent advantage, i.e., such resonators are etched in the substrate side opposite to that of the line strip. Consequently, these sensors provide backside isolation, and the presence of the MUT is in contact, or in close proximity, to the sensing resonators does not alter the propagation along the line (except by the fact that the MUT modifies the resonance frequency of the sensing element). Thus, let us consider sensor implementation by means of slot-type resonators, such as the dumbbell defect-ground-structure (DB-DGS) resonator [53], the complementary split-ring resonator (CSRR) [54], and the open complementary split-ring resonator (OCSRR) [55], to name a few.

Let us consider that the sensors under study are devoted to permittivity measurements. The canonical input and output variables are thus the dielectric constant of the material under study (or material under test $\varepsilon_{\mathrm{MUT}}$) and the resonance frequency, respectively. Nevertheless, if the imaginary part of the complex dielectric constant (or the loss tangent) should also be retrieved (second input variable), then an additional output variable is required. Such a variable is typically the magnitude of the notch (or peak, in case of bandpass-type sensing structures) [18,56,57,58]. The canonical sensitivity is thus defined as the derivative of the resonance frequency of the sensing element, $f_{0}$, with respect to the dielectric constant of the material under test (MUT), $\varepsilon_{\mathrm{MUT}}$, i.e.,

(1) $$S=\frac{df_{0}}{d\varepsilon_{\mathrm{MUT}}}$$

It is obvious that the variation in the resonance frequency generated by a change in the dielectric constant of the MUT should be larger in high-frequency sensors. Thus, for a faithful comparison between the sensitivities of different frequency-variation sensors, the relative frequency change is the one that should be considered. Therefore, the relative sensitivity, defined as

(2) $$\bar{S}=\frac{1}{f_{0}}\;\cdot \;\frac{df_{0}}{d\varepsilon_{\mathrm{MUT}}}$$

is the relevant parameter and figure of merit (useful for a realistic comparison) in these frequency-variation sensors. Assuming that the MUT is in the region of influence of the resonant element, a variation in the dielectric constant of the MUT alters the capacitance of such resonant element, and this in turn produces a shift in the resonance frequency. Thus, the relative sensitivity can be further developed and expressed as

(3) $$\bar{S}=\frac{1}{f_{0}}\;\cdot \;\frac{df_{0}}{dC^{\prime }}\;\cdot \;\frac{dC^{\prime }}{d\varepsilon_{\mathrm{MUT}}}$$

where $C^{\prime }$ is the capacitance of the resonant element loaded with the MUT. The first derivative in (3) depends on the specific configuration of the line and sensing resonator, i.e., it depends on the analytical dependence of $f_{0}$ with $C^{\prime }$. The second derivative in the relative sensitivity can be analytically obtained provided that several hypothesis and approximations are adopted [52]. The first assumption considers the analysis restricted to fully planar resonant sensing elements etched in a single metallic layer and, therefore, exhibiting an edge capacitance. Namely, resonant elements implemented by means of two metallic layers, such as the broadside-coupled split-ring resonator (BC-SRR), or the microstrip step-impedance shut stub (SISS) resonator, both exhibiting a broadside capacitance, are excluded in the present analysis. It is also considered that the metal is an ideal (perfect) conductor, with negligible resistivity and thickness (this approximation is very reasonable in the usual metallization present in common microwave substrates, typically made of a high-conductivity material, such as Cu, and with thicknesses of the order of few tens of microns). The MUT is considered to be in contact with the resonant element, and occupying the whole half-space. That is, it is semi-infinite in the vertical direction (i.e., the direction orthogonal to the plane of the resonator), with transverse dimensions extending beyond the region occupied by the resonator. Moreover, the MUT is made of a uniform material. The substrate (also uniform) is sufficiently thick so that the effects of any potential metallic pattern in the face opposite to the one where the resonator is etched (e.g., the line strip) can be neglected (i.e., the resonance frequency is not altered by the presence of such patterns).

Let us consider a slot resonator etched in the ground plane of a microstrip line, and let us neglect the effects of the line on the capacitance (and resonance) of the resonator. If the substrate is thick enough to be considered semi-infinite in the vertical direction, it follows that the plane of the resonant element is a magnetic wall, and the electric field distribution at both half-spaces is a mirror image [59]. Under these conditions, the electric field in the plane of the resonator is tangential, and the capacitance of the resonator can be separated in two parts, i.e., the capacitances associated with the lower and upper half-spaces (representing the contributions of the MUT and substrate, respectively), both connected in parallel. If we designate by C the capacitance of the bare resonator, i.e., without MUT (or surrounded by air), the capacitance $C^{\prime }$ of the resonator loaded with an arbitrary MUT can be expressed as [18,52,57,58,59]

(4) $$C^{\prime }=C\frac{\varepsilon_{r}+\varepsilon_{\mathrm{MUT}}}{\varepsilon_{1}+1}$$

where $\varepsilon_{r}$ is the dielectric constant of the substrate. According to (4), the second derivative in (3) is found to be

(5) $$\frac{dC^{\prime }}{d\varepsilon_{\mathrm{MUT}}}=\frac{C}{\varepsilon_{r}+1}$$

**Figure 1:**
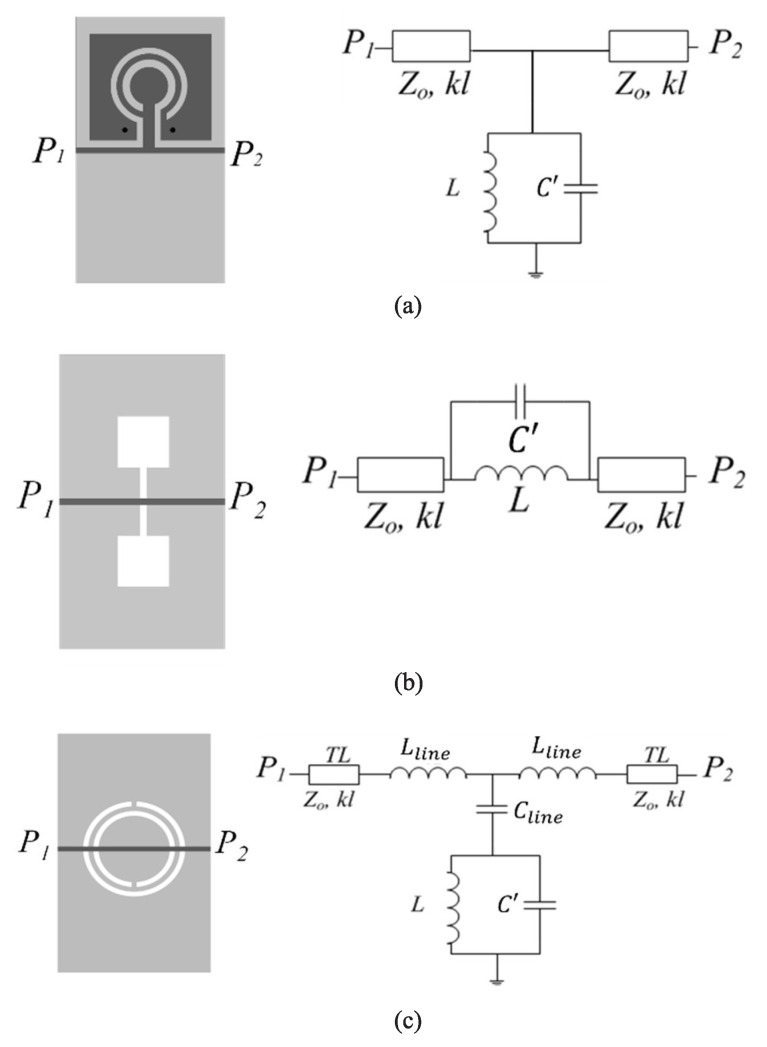
Typical topology and circuit model of a microstrip line loaded with a CSRR (**a**), DB-DGS resonator (**b**), and OCSRR (**c**). $Z_{0}$ and kl are the characteristic impedance and electrical length, respectively, of the host lines at both sides of the resonator (*k* and *l* being the phase constant and length, respectively). Reproduced with permission from [59].

Concerning the first derivative in (3), as indicated, it depends on the specific sensor configuration and resonant element. Let us consider three different resonant elements, the CSRR, the DB-DGS, and the OCSRR, loading a microstrip line, as depicted in Figure 1. The circuit models of these structures are also depicted in Figure [18,39,53,60,61]. It is clear that, for the OCSRR and DB-DGS resonators, the dependence of the resonance frequency on $C^{\prime }$ is simply

(6) $$f_{0}=\frac{1}{2\pi \sqrt{LC^{\prime }}}$$

whereas for the CSRR, the following result is obtained

(7) $$f_{0}=\frac{1}{2\pi \sqrt{L(C_{line}+C^{\prime })}}$$

where *L* is the inductance of the resonator, not affected by the MUT, and $C_{line}$ is the capacitance of the line section on top of the CSRR.

Using (7), it follows that

(8) $$\frac{df_{0}}{dC^{\prime }}=-\frac{1}{2}\;\cdot \;\frac{f_{0}}{C_{line}+C^{\prime }}$$

and introducing (8) and (5) in (3), the relative sensitivity for the CSRR-loaded line is found to be

(9) $$\bar{S}=-\frac{1}{2}\;\cdot \;\frac{C}{C_{line}\left(\varepsilon_{r}+1\right)+C\left(\varepsilon_{r}+\varepsilon_{\mathrm{MUT}}\right)}$$

It can be easily deduced that the relative sensitivity for the OCSRR- and the DB-DGS-loaded microstrip lines can be inferred from (9) by simply forcing $C_{line}$ = 0. From these results, it can be concluded that the relative sensitivity is superior in OCSRR- and DB-DGS-loaded microstrip lines, as compared to the CSRR-loaded lines. Moreover, from (9 with $C_{line}$ = 0), it follows that the relative sensitivity in sensors based on OCSRR and DB-DGS resonators does not depend on the geometry of the resonant element. However, note that the assumption of a semi-infinite MUT and substrate has been considered. Concerning the MUT, it is possible to machine it in order to be thick enough so that the semi-infinite approximation holds. In contrast, the substrate thickness is sometimes determined by external factors that do not depend on the designer, and, therefore, the semi-infinite substrate approximation cannot always be guaranteed. Despite that fact, even for relatively thin substrates, the plane of the resonant element is a quasi-magnetic wall [59]. Under these circumstances, the capacitance of the resonator can still be considered to be formed by the parallel connection of the capacitance of the MUT and the capacitance of the substrate. Such capacitance is given by an expression formally identical to (4), but replacing $\varepsilon_{r}$ with $\varepsilon_{r,eq}$, the latter being the equivalent dielectric constant of the substrate, defined as the dielectric constant of a hypothetical semi-infinite substrate providing the same contribution to the resonator’s capacitance [59,62]. This means that, for a finite substrate, the relative sensitivity for the CSRR-loaded line should be rewritten as

(10) $$\bar{S}=-\frac{1}{2}\;\cdot \;\frac{C}{C_{line}\left(\varepsilon_{r,eq}+1\right)+C\left(\varepsilon_{r,eq}+\varepsilon_{\mathrm{MUT}}\right)}$$

**Figure 2:**
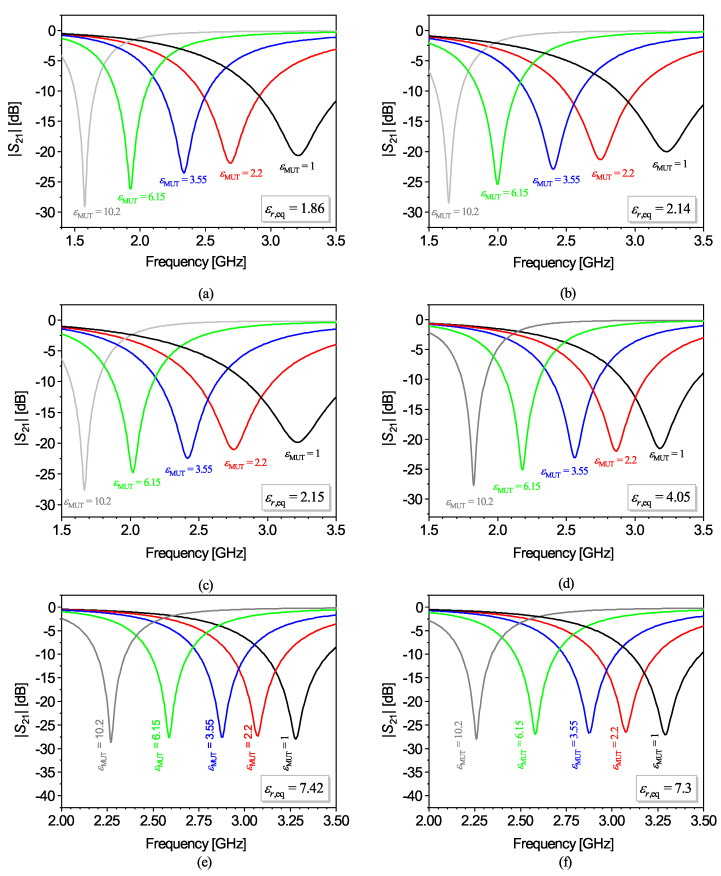
Responses of the sensors for different dielectric constants of the MUT (indicated). (**a**) Sensor A; (**b**) sensor B; (**c**) sensor C; (**d**) sensor D; (**e**) sensor E; and (**f**) sensor F. Reproduced with permission from [59].

**Figure 3:**
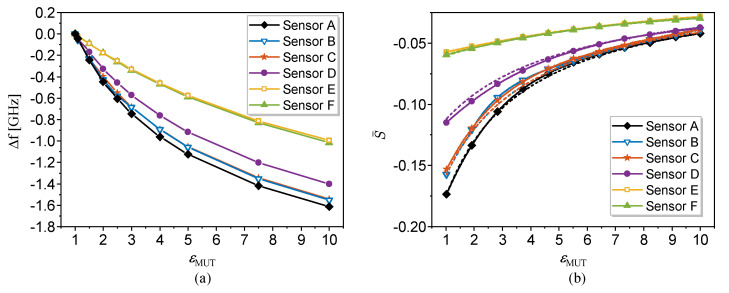
Variation of the resonance frequency with the dielectric constant of the MUT (**a**) and relative sensitivity (**b**) for sensors A, B, C, D, E and F. The theoretical relative sensitivity inferred from expression (10) is also included in (**b**), in dashed line. Reproduced with permission from [59].

The equivalent dielectric constant depends on the ratio between the width of the capacitive slot and the thickness of the substrate [59]. Consequently, for finite substrates, i.e., for substrates not satisfying the semi-infinite approximation, the relative sensitivity in OCSRR- or DB-DGS-loaded microstrip lines, given by (10), does depend on the geometry. However, the dependence is soft, especially for relatively thick substrates, as it has been recently demonstrated [59] (also note that, as the substrate thickness increases, the equivalent dielectric constant tends towards the nominal dielectric constant of the substrate, or $\varepsilon_{r,eq}\to \varepsilon_{r}$). Nevertheless, it can be concluded that regardless of the specific structure, for sensitivity optimization, it is convenient to deal with low dielectric constant substrates. In addition, it can be concluded from (9) and (10) that, as the dielectric constant of the MUT increases, the relative sensitivity decreases. Indeed, for high dielectric constant MUTs, the sensitivity scarcely depends on $\varepsilon_{r}$ (or $\varepsilon_{r,eq}$), since $\varepsilon_{\mathrm{MUT}}$ obscures the substrate dielectric constant (or equivalent dielectric constant). In [63], it was demonstrated that the relative sensitivity in an OCSRR- and DB-DGS-based sensor was very similar, and superior to the one obtained in a CSRR-based sensor (all implemented in the same substrate), in agreement with the theory. In this review paper, let us reproduce the results concerning the relative sensitivity achieved in various sensing structures based on a microstrip line loaded with a DB-DGS resonator ($W_{s}$ is the strip width, whereas the DB-DGS is characterized by the width *S* and length *l* of the slot, and the width $W_{a}$ and length $l_{a}$ of the apertures, square-shaped in all cases), as can be seen in Table 1. The responses for MUTs with different dielectric constants (all semi-infinite in the vertical direction), inferred from electromagnetic simulation, are depicted in Figure 2 [59], where the equivalent dielectric constant is indicated. Figure 3a depicts the dependence of the resonance frequency with the dielectric constant of the MUT for the different sensors, whereas Figure 3b depicts the relative sensitivity. The agreement between the relative sensitivity inferred from the derivative of the simulated data points and the analytical expression (10) is excellent. Moreover, it can be appreciated from Figure 2b that those sensors exhibiting the same (or roughly the same) equivalent dielectric constant exhibit an indistinguishable relative sensitivity, further pointing out the validity of the analysis.

In order to obtain the dielectric constant of an unknown material (MUT), isolating it from (4) is the first step, which gives

(11) $$\varepsilon_{\mathrm{MUT}}=\left(\varepsilon_{r,eq}+1\right)\frac{C^{\prime }}{C}-\varepsilon_{r,eq}$$

Note that, in (11), we replaced the dielectric constant of the substrate by the equivalent dielectric constant in order to take into account the finite thickness of the substrate. If the considered resonator is the DB-DGS (or the OCSRR), with the resonance frequency given by (6), then (11) can be rewritten in terms of the resonance frequency of the bare resonator, $f_{0,\mathrm{air}}$, and in terms of the resonance frequency of the resonator loaded with the MUY, $f_{0,\mathrm{MUT}}$, i.e.,

(12) $$\varepsilon_{\mathrm{MUT}}=\left(\varepsilon_{r,eq}+1\right)\frac{f_{0,\mathrm{air}}^{2}}{f_{0,\mathrm{MUT}}^{2}}-\varepsilon_{r,eq}$$

For CSRR-based sensors, the ratio $C^{\prime }/C$ that appears in (11) can be evaluated by isolating *C* and $C^{\prime }$ from (7), however, the resulting expression is not as simple as (12). In this case, it is found that $C^{\prime }/C$ is

(13) $$\frac{C^{\prime }}{C}=1-\frac{1}{2\pi^{2}LC}\left\{\frac{1}{f_{0,\mathrm{air}}^{2}}-\;\frac{1}{f_{0,\mathrm{MUT}}^{2}}\right\}$$

and the dielectric constant of the MUT is inferred by introducing (13) in (11). Another important aspect is the determination of the equivalent dielectric constant of the substrate. For this purpose, the idea is to use (12), for DB-DGS (or OCSRR) based sensors, and consider a MUT with well-known dielectric constant. By measuring, or simulating, the response of the bare sensor, and the one of the sensor loaded with that MUT, $\varepsilon_{r,eq}$, can be isolated from (12). If, in contrast, a CSRR is the sensing element, in this case, (13) and (11) must be used. The experimental validation of frequency-variation permittivity sensors based on different resonant elements, such as CSRRs, SRRs or DB-DGS resonators, has been reported in the literature [18,58]. Table 2 depicts a comparison of various frequency-variation sensors. In the table, $f_{0,b}$ is the frequency of the bare resonator. ${\bar{S}}_{av}$ is the average sensitivity, calculated as the ratio between the output dynamic range (or difference between the resonance frequency of the bare resonator and the resonance frequency of the resonator loaded with the MUT exhibiting the maximum dielectric constant) and the input dynamic range (difference between the maximum and minimum values of $\varepsilon_{\mathrm{MUT}}$). Finally, ${\bar{S}}_{av}$ is the relative average sensitivity, calculated by simply dividing the average sensitivity by the resonance frequency of the bare resonator, i.e., ${\bar{S}}_{av}=S_{av}/f_{0,\mathrm{air}}$) (nevertheless, note that in the table, ${\bar{S}}_{av}$ is expressed as a percentage). The table includes also the considered input dynamic range relative to the dielectric constant of the MUT.

According to the results of Table 2, the sensor reported in [58] exhibits by far the higher relative average sensitivity. It should be mentioned that the substrate dielectric constant in the sensors of [64,66] is $\varepsilon_{r}=10.2$, and this explains, in part, the limited relative average sensitivity in such sensors. However, in the sensors reported in [28,65], the dielectric constant of the substrate is $\varepsilon_{r}=2.94$ and $\varepsilon_{r}=2.33$, i.e., smaller than the substrate dielectric constant considered in the sensor of [58] ($\varepsilon_{r}=3.55$). The reason that explains the superior relative average sensitivity in the sensor of [58] is the considered resonator, a DB-DGS. With such a sensing resonant element, the changes in the dielectric constant of the MUT directly affect the unique capacitance that determines the resonance frequency, the output variable (see expression (6)). In contrast, in the sensors presented in [28,65], where a complementary split-ring resonator (CSRR) was used, the resonance frequency is also dependent on the coupling capacitance between the line and the resonant element ($C_{line}$), as can be seen in the expression (7). Since such capacitance does not vary with the dielectric constant of the MUT, it is expected that such CSRR-based sensors exhibit poorer sensitivity, as compared to those of DB-DGS-based sensors.

### 2.2. Exploiting the Coupling between Resonators

Recently, it was shown that the sensitivity of CSRR-based sensors can be considerably enhanced by loading multiple CSRRs to a microstrip line (or antenna) and exploiting the inter-resonator coupling between them [33,34,67]. In [33], it was theoretically and experimentally demonstrated that when the resonators are placed such that their coupling takes place in the plane transverse to the direction of propagation (along with the microstrip line), the sensitivity of the sensor enhances. It was also shown that increasing the number of resonators creates more sensitive modes (transmission zeros). In [34,67], highly sensitive sensors based on coupled CSRRs were applied for sensing glucose levels in the blood and aqueous solutions.

**Figure 4:**
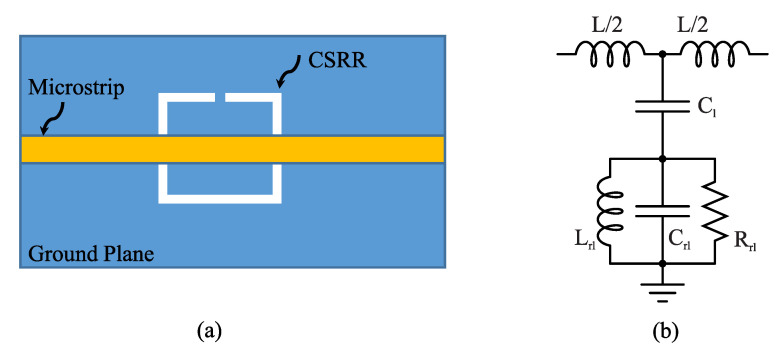
A microstrip line loaded with a CSRR: (**a**) footprint and (**b**) the equivalent lumped circuit model. Reprinted with permission from Ref. [33].

Let us first study how the mutual coupling between resonators can enhance the sensitivity of a CSRR-based sensor [33]. Figure 4a shows a microstrip line loaded with a single CSRR. The CSRR can be considered as a quasi-static resonator and modeled using an RLC circuit as shown in Figure 4b. In this figure, $C_{1}$ and *L* are the per unit-length capacitance and inductance of the transmission line and $C_{r1}$ and $L_{r1}$ are the effective capacitance and inductance of the resonator. The circuit shown in Figure 4b exhibits a transmission of zero at its resonance frequency given by

(14) $$f_{z0}=\frac{1}{2\pi \sqrt{L_{r1}\left(C_{r1}+C_{1}\right)}}$$

**Figure 5:**
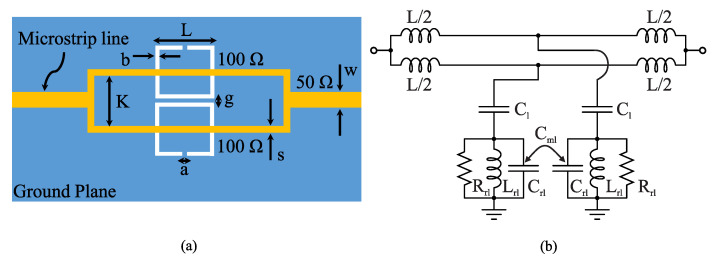
A splitter–combiner microstrip section loaded with two-coupled CSRRs (**a**) footprint (a=b=g=0.5 mm, L=7.5 mm, and K=7.634 mm) and (**b**) equivalent lumped circuit model. Reprinted with permission from Ref. [33].

Loading the CSRR with a dielectric material increases the resonator’s capacitance ($C_{r1}$) due to the higher unity of the sample’s relative permittivity, which decreases the resonance frequency. In Figure 5a, loading a microstrip line with two CSRRs is considered. For stronger coupling between the line and CSRRs, the resonators are individually excited using two branches of the transmission line in the form of a splitter–combiner microstrip Section [68]. Figure 5b shows the lumped circuit model for the splitter–combiner microstrip section loaded with two CSRRs [68]. In this figure, $C_{m1}$ is the mutual capacitance modeling the inter-resonator coupling between two CSRRs. Notice that since the resonators are electrically very close to each other, the inter-resonator coupling is appreciable. Considering that the coupling between the two transmission line sections is very low, the resonance frequency (in which a transmission zero occurs) can be approximated by [68],

(15) $$f_{z}=\frac{f_{z0}}{\sqrt{1-\frac{C_{m1}}{C_{r1}+C_{1}}}}$$

where $f_{z0}$ is the resonance frequency without the inter-resonator coupling between the CSRRs given in (14). From (15), the inter-resonator coupling ($C_{m1}$) causes an increase in the resonance frequency.

To analyze the sensitivity of the 1CSRR sensor compared to that of the 2CSRR sensor, the assumption of a small change in the resonance frequency due to the perturbation caused by the MUT was made. Since the MUT mostly affects the resonator capacitance ($C_{r1}$), the derivative of the resonance frequency with respect to $C_{r1}$ can be considered as a measure of the sensitivity. For the 1CSRR sensor, in (14), we have

(16) $$\frac{df_{z0}}{dC_{r1}}=-\frac{L_{r1}}{4\pi {\left(L_{r1}\left(C_{r1}+C_{1}\right)\right)}^{\frac{3}{2}}}=-\frac{f_{z0}}{2\left(C_{r1}+C_{1}\right)}$$

whereas for the 2CSRR sensor, in (14), we have

(17) $$\begin{array}{cc} \frac{df_{z}}{dC_{r1}} & =-\frac{L_{r1}}{4\pi {\left(L_{r1}\left(C_{r1}+C_{1}\right)\right)}^{\frac{3}{2}}{\left(1-\frac{C_{m1}}{C_{r1}+C_{1}}\right)}^{\frac{3}{2}}}\\ \; & =-\frac{f_{z}}{2\left(C_{r1}+C_{1}\right)\left(1-\frac{C_{m1}}{C_{r1}+C_{1}}\right)}. \end{array}$$

It can be seen that compared to the changes in the resonance frequency of the 1CSRR sensor (i.e., (16)), the inter-resonator coupling capacitance ($C_{m1}$) in (17) makes the resonance frequency of the 2CSRR sensor more sensitive to the change in the resonator capacitance ($C_{r1}$); hence, a higher sensitivity to the change in the MUT can be achieved.

**Figure 6:**
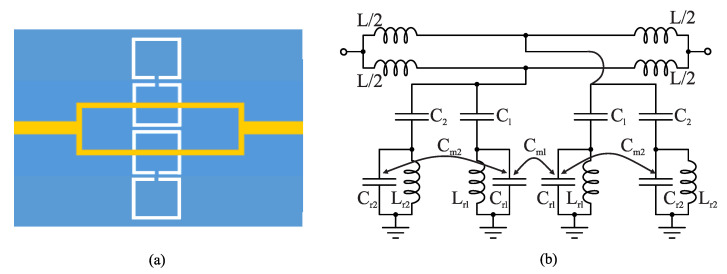
A splitter–combiner microstrip section loaded with a four-coupled CSRRs (**a**) footprint and (**b**) equivalent lumped circuit model. Reprinted with permission from Ref. [33].

To further increase the sensitivity, four resonators can be coupled to two transmission lines as shown in Figure 6a. Figure 6b shows the lumped circuit model in the case of the four resonators. Since the two feeding transmission lines are not in the center islands of the two additional resonators, it is expected that their coupling capacitance ($C_{2}$) becomes weaker. The new mutual coupling can be represented as an inter-resonator capacitance ($C_{m2}$). In addition, since we have two sets of coupling capacitances ($C_{1}$ and $C_{2}$) as well as two inter-resonator mutual coupling capacitances ($C_{m1}$ and $C_{m2}$), it is expected that the sensor exhibits a dual-band rejection (i.e., two transmission zeros).

**Figure 7:**
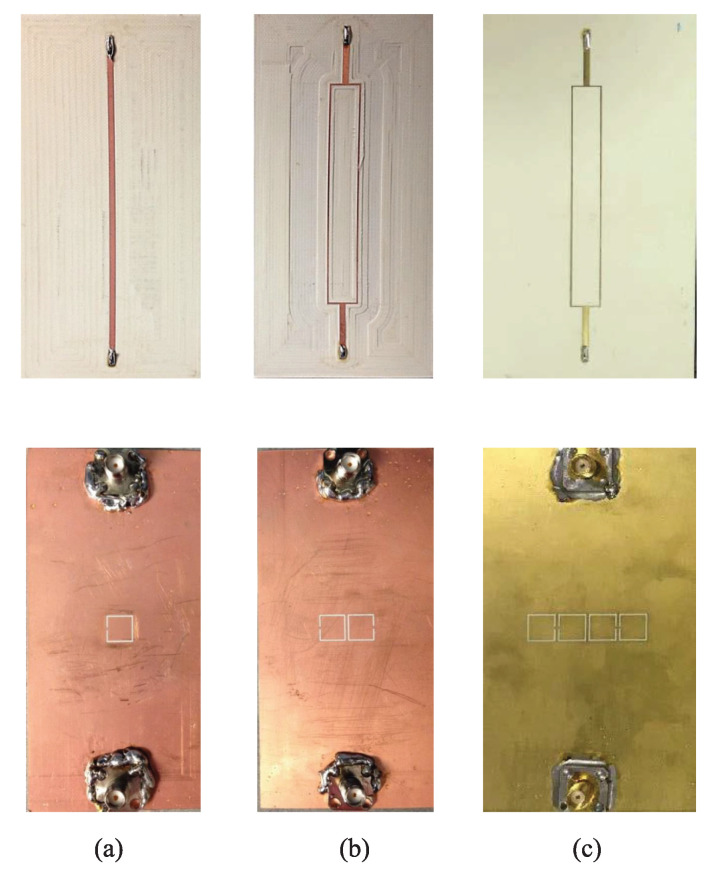
The top and bottom views of the fabricated 1CSRR (**a**), 2CSRR (**b**) and 4CSRR (**c**) sensors. Reprinted with permission from Ref. [33].

**Figure 8:**
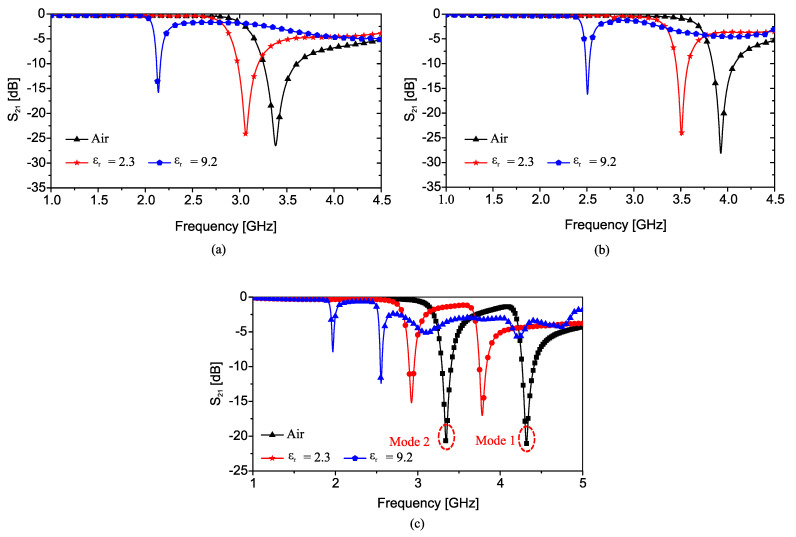
Experimentally measured transmission coefficient (|*S*${}_{21}|$) of the fabricated 1CSRR (**a**), 2CSRR (**b**) and 4CSRR (**c**) sensors. Reprinted with permission from Ref. [33].

**Figure 9:**
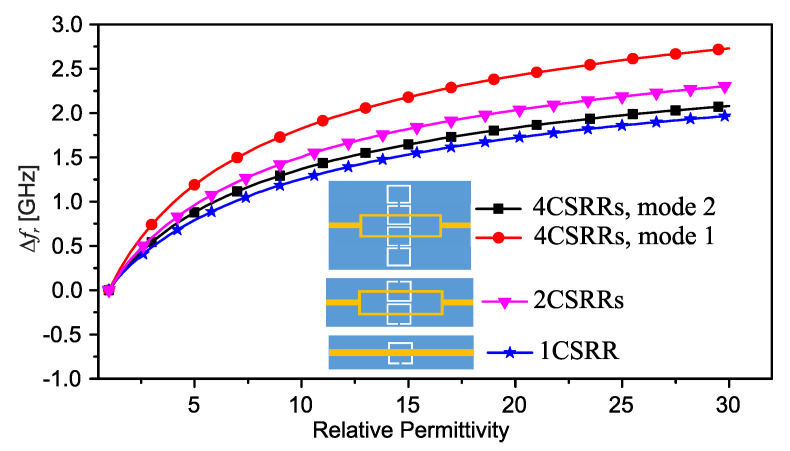
Resonance frequency shifts versus the dielectric constant of the slab that is varied from 1 to 30 with a step size of 0.2. Reprinted with permission from Ref. [33].

As a proof of concept, the authors of [33] designed a 50 Ω microstrip line loaded with a CSRR on a Rogers RO4350 laminate with a thickness of 0.75 mm such that the resonance frequency (transmission zero) occurs at approximately 3.4GHz. They also designed the 2CSRR and 4CSRR sensors so that each CSRR’s dimensions are the same as that in the 1CSRR sensor. The sensors were fabricated as shown in Figure 7. The measured transmission coefficient (|*S*${}_{21}|$) parameters of the sensors when unloaded as well as when loaded with Rogers RT/duroid 5870 and TMM10 laminates having dielectric constants of 2.3 and 9.2 are shown in Figure 8. As seen in this figure, the resonance frequency of the unloaded 2CSRR sensor is approximately 3.9 GHz, i.e., approximately 500 MHz higher than the resonance frequency of the 1CSRR sensor, which agrees with the analysis of the equivalent circuit models. The 4CSRR sensor, as expected from its equivalent circuit model, also shows two transmission zeros (resonances) at approximately 4.3 GHz (first mode) and 3.4 GHz (second mode). Compared to the reference case (unloaded sensors), the shifts in the resonance frequency when loaded with RT/duroid 5870 and TMM10 laminates are 313 MHz and 1243 MHz for the 1CSRR sensor, 420 MHz and 1420 MHz for the 2CSRR sensor, and 515 MHz and 1742 GHz for the first mode of the 4CSRR sensor. Moreover, for a better comparison between the sensitivity of three sensors, in the full-wave simulations, the dielectric constant of an MUT (filling the CSRRs’ area) was varied from 1 to 30. Figure 9 shows the shifts in the resonance frequencies of the 1CSRR, the 2CSRR and the 4CSRR sensors. Figure 8 and Figure 9 demonstrate that, as expected from the analysis of the equivalent lumped circuits, the 2CSRR sensor shows appreciably higher sensitivity than the 1CSRR sensor. In addition, in the case of the 4CSRR sensor, mode 1 shows much higher sensitivity to changes in the dielectric constant of the MUT. This validates the concept of exploiting the coupling between resonators to enhance sensitivity.

**Figure 10:**
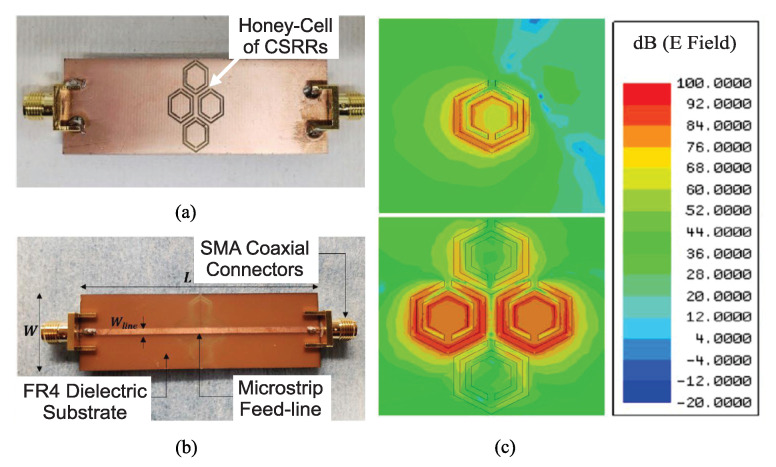
Honey-cell CSRR sensor consisting of four coupled hexagonal-shaped CSRRs (**a**) coupled to a microstrip line (**b**). Electric field distribution on the CSRR surface at a 3.0 GHz resonant frequency when a honey-cell and a single hexagonal-shaped CSRR are used. (**c**) Electric field concentration at the frequency of resonance for both single (top) and coupled CSRRs (bottom). Reprinted with permission from Ref. [34].

In [34], as shown in Figure 10a,b, four hexagonal-shaped CSRRs, arranged in a honey-cell configuration and coupled to a microstrip line, were applied for monitoring the blood glucose level. Due to the resonant feature of the CSRRs, transmission zeros appear at the $S_{21}$ of the line. Figure 10c indicates a comparison between the electric field distributions (at a 3 GHz resonant frequency) on the ground plane when a honey-cell (including four coupled hexagonal CSRRs) and a single hexagonal-shaped CSRR are etched on the ground plane. It can be seen that the honey-cell design exhibits higher electric field localization with an intensity up to $10^{5}\;V/m$ over the CSRR area compared to that of a single hexagonal cell. Due to the high intensity of the electric field coupled to the honey-cell area (around the dielectric slits and the routes in-between), this area is identified as the sensing region for the glucose samples to acquire strong interaction with the coupled near-field and therefore induce notable variations in the intrinsic characteristics of the sensor in response to subtle variations in the EM properties of different glucose concentrations.

**Figure 11:**
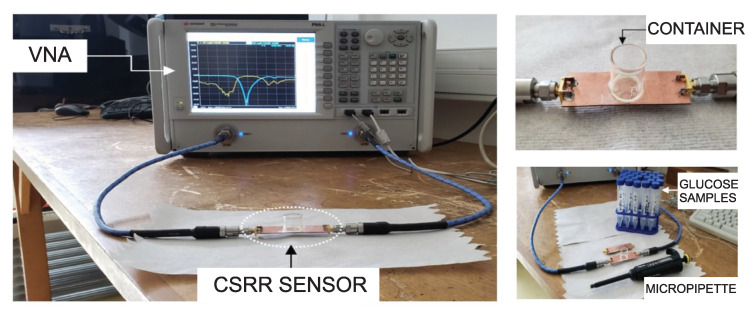
Glucose-sensing experiments: VNA experimental setup, sensor loaded with an empty container and the micropipette and glucose aqueous solutions used in the experiments. Reprinted with permission from Ref. [34].

Two preliminary honey-cell prototypes (compact and dispersed) were numerically modelled, fabricated and experimented with for monitoring the glucose level. Figure 11 shows the experimental setup using a vector network analyzer (VNA). For the sake of simplicity, aquatic glucose solutions were used in these experiments to imitate the blood behaviour at different glucose concentrations (70–120 mg/dL) clinically relevant to type-2 diabetes. This approximation is valid since water contributes approximately 50% of the entire human blood that contains other vital components at varying proportions. These minerals are present at lower concentrations compared to the dominant glucose, whereby the blood dielectric properties are dominantly affected. A cylindrical glass container was fabricated to hold the samples on top of the CSRR surface (see Figure 11). The empty cylindrical container was placed on the honey-cell CSRR structure, as shown in Figure 4b. This will introduce a few MHz shifts from the reference resonance in *S*_21_ of the unloaded state. In each measurement trial, a micropipette device was used to measure a precise volume of V=600μL from each concentration, load it inside the container, and the change in the transmission resonance frequency response was recorded.

**Figure 12:**
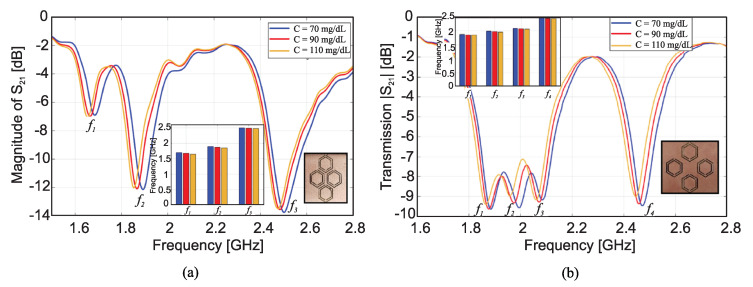
Measured transmission response $S_{21}$ as function of frequency for the tested glucose samples of various concentrations for (**a**) compact and (**b**) dispersed sensors. Reprinted with permission from Ref. [34].

**Figure 13:**
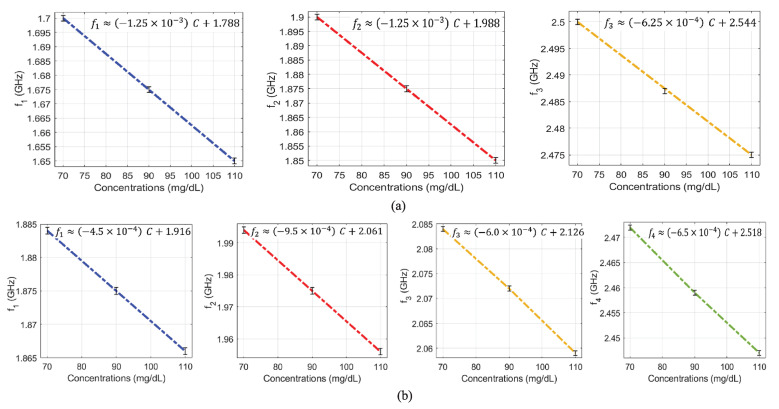
Linear correlation models for the resulting resonant frequencies of (**a**) compact sensor at three frequencies of $f_{1}$ (in blue), $f_{2}$ (in red), and $f_{3}$ (in orange), and (**b**) dispersed sensors at four frequencies of $f_{1}$ (in blue), $f_{2}$ (in red), $f_{3}$ (in orange), and $f_{4}$ (in green) at different glucose concentrations. Reprinted with permission from Ref. [34].

Figure 12a,b, respectively, show the transmission responses of the compact and dispersed sensors when the concentration of glucose is changing in 70, 90 and 110 mg/dL. In the compact prototype, as shown in the inset of Figure 12a, the CSRRs are located close together; hence, there is a strong mutual coupling between them. Whereas in the dispersed prototype indicated in the inset of Figure 12b, the CSRRs are spaced apart, and the mutual coupling is weak. In these figures, three and four transmission zeros are observed in the response of the compact and dispersed sensors, respectively. In both sensors’ responses, it is observed that the resonant frequencies at which the transmission is minimized are shifted towards lower frequencies as the glucose concentration in the sample increases. Linear correlation models for the resultant resonant frequencies of compact and dispersed sensors at different glucose concentrations were derived, which are shown in Figure 13a,b, respectively (conversely, the inverse models could be used to estimate the unknown glucose level of a tested sample). It is observed that the respective resonant frequencies decrease with increasing levels of glucose concentrations. However, the frequency resolutions for glucose level changes at the respective resonances are not identical. The sensitivity of the compact sensor is estimated as −1.25/(mg/dL), representing the gradient of $f_{1}$ and $f_{2}$ linear models. However, the best sensitivity slope for the dispersed topology (where the mutual coupling between the resonators is weak) is recorded as −0.95/(mg/dL) at $f_{2}$, which is lower than the compact counterpart having a strong coupling between its CSRRs. This demonstrates the positive impact of mutual coupling on sensitivity.

**Figure 14:**
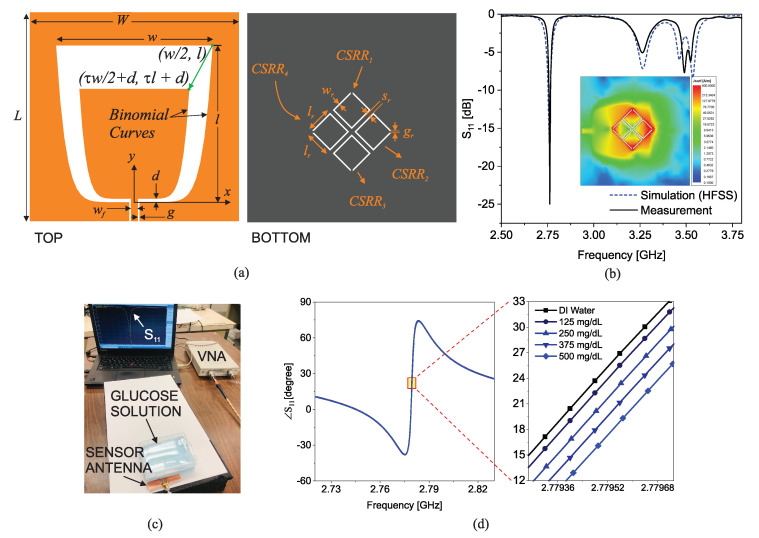
Structure of the fabricated sensor antenna. (**a**) Schematic of sensor antenna, $l=w=45,\;N=8,\;\tau =0.7,\;d=1,\;w_{f}=2,\;W=L=60,\;l_{r}=7.8,\;s_{r}=1,\;g=g_{r}=w_{r}=0.5$ (all lengths are in (mm)); (**b**) comparison between simulated and measured $|S_{11}|$ with inset simulated surface current density at 2.78 GHz; (**c**) measurement setup; and (**d**) the phase response for various glucose concentrations. Reprinted with permission from Ref. [67].

In another similar recent work [67], a planar sensor antenna consisting of coupled CSRRs excited by a binomial slot antenna was introduced for glucose sensing. A schematic of the proposed sensor antenna is shown in Figure 14a. A wide-band CPW-fed printed slot antenna with a binomial curve, as shown on the left side of the figure, is designed on the top of a dielectric substrate to have a good matching within 2.6–3.6 GHz bandwidth. On the bottom of the substrate, as shown on the right side of Figure 14a, four identical CSRRs are etched.

The measured and simulated responses ($|S_{11}|$) of the sensor antenna are shown in Figure 14b indicating a good agreement between them. A deep resonance is observed at 2.78 GHz which is not produced by only one CSRR but is uniquely formed by the mutual interaction of four of them as can be verified by the surface current density given in the inset over all resonators. In fact, the objective of this work was to enhance the sensitivity by exploiting the mutual coupling between the four resonators. The phase-response of the sensor at the lowest resonance frequency (i.e., 2.78 GHz) was used to sense the glucose level in aqueous solutions due to its high sensitivity. Notably, the dielectric constant of the aqueous solution decreases at a higher glucose content, thereby increasing the resonance frequency. This upshift leads to a drop in the phase when monitored at a single frequency. As a proof of concept, four samples of aqueous solution with 125 mg/dL increments were prepared. As shown in Figure 14c, the samples were held 5 mm above the sensor (using foam) within a plastic container to emulate the thickness of skin, fat and muscle layers. The phase response of the sensor was measured, as shown in Figure 14d. This figure shows that the phase at 2.78 GHz changes from 25.4 to 17.2 degrees for a glucose level change of 0–500 mg/dL, with a sensitivity beyond 0.5 degrees corresponding to 30 mg/dL.

### 2.3. Embedded Channels in the Substrate

**Figure 15:**
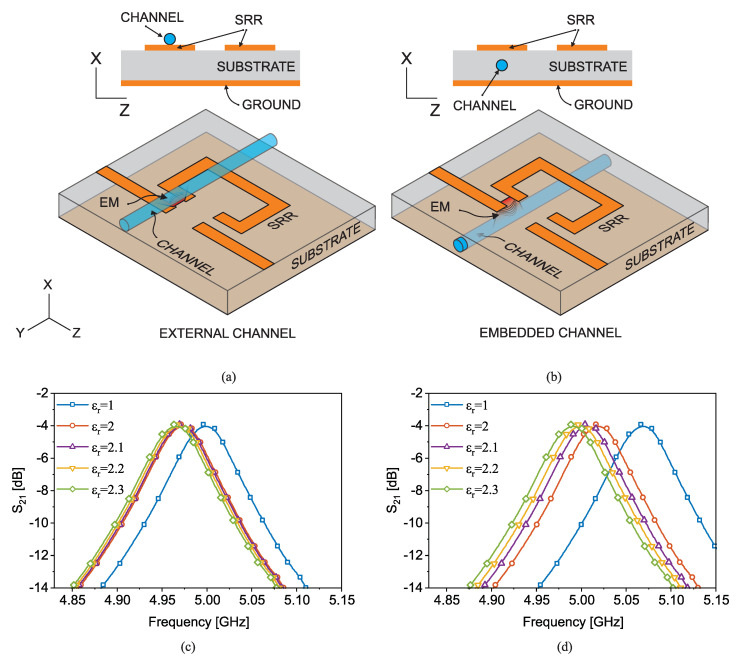
Microwave sensor with a tube (**a**) above and (**b**) below the sensor. (**c**) Simulation results with the sensor being loaded by a dielectric material with different permittivity values inside (**c**) an external channel and (**d**) an internal channel. Reprinted with permission from Ref. [36].

In this section, the substrate of a microwave sensor is shown that can be exploited as the analyte carrier to boost the sensor sensitivity. Microwave sensors are typically designed on low-permittivity substrates as the base of the sensor. This low-permittivity is essentially because of the overall interaction of a substrate with the sensitivity. To elaborate on this, let us consider a planar half-wavelength (λ/2) resonator (open to free space) that is supported by a dielectric substrate backed by a ground plane as shown in Figure 15. The sensor’s resonance frequency is governed by:

(18) $$f_{0}=\frac{1}{2\pi \sqrt{L_{eq}C_{eq}}}$$

where $L_{eq}$ and $C_{eq}$ denotes the effective inductance and capacitance seen by the resonator including the substrate and background materials in the proximity of the resonator ($\varepsilon_{r}⩾1$). Then, any change in a dielectric material under test results in capacitance variation is as follows:

(19) $$C_{eq}=C_{0}+C_{\mathrm{MUT}}=C_{0}\left(1+\frac{C_{\mathrm{MUT}}}{C_{0}}\right)$$

where $C_{0}$ is the capacitance of a bare resonator without the presence of any external interfering material, and $C_{\mathrm{MUT}}$ represents the whole impact of an external analyte. This expression suggests that the resonance frequency shifts according to a percentage change in the overall capacitance of a resonator. Therefore, a high sensitivity can be achieved by increasing the ratio of $C_{\mathrm{MUT}}/C_{0}$. As a result, the substrate that is a contributing factor for $C_{0}$ needs to be chosen to be low as not to obscure the material’s impact. In this regard, since typically $C_{0}\gg C_{\mathrm{MUT}}$, one can exploit the very main contributing factor rising from the substrate. In such a case as that shown in detail in [36], the substrate can be used as the carrier of the material as opposed to a case where the MUT is separate from the substrate with a minimal impact on modifying the total capacitance $C_{eq}$. By properly embedding the material inside the substrate, $C_{0}$ changes significantly since the majority of electric fields between the resonator and the ground plane become disturbed. According to the diagram shown in Figure 15a, conventional placement of the channel is outside the resonator flushed with the sensor surface when exposing the material to the sensor with shortest possible distance. The electric fields interacting with the MUT are emanating fields fringing from the transmission line into air and terminating at the resonator. In contrast, the method studied in [36] is represented in Figure 15b in detail where the microfluidic channel is embedded within the substrate. This configuration allows for higher interaction between MUT and the resonator-to-ground electromagnetic fields, which leads to higher sensitivity.

**Figure 16:**
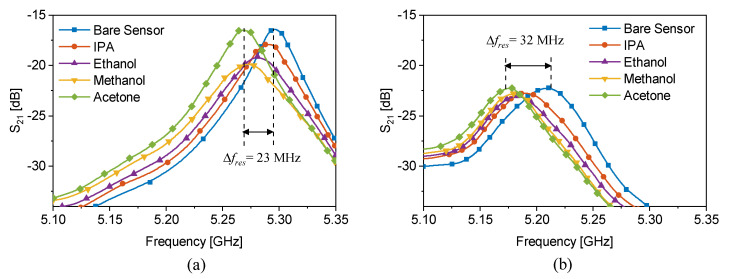
Measured transmission response for both sensors using (**a**) external channel and (**b**) embedded channel filled with IPA, ethanol, methanol and acetone. Reprinted with permission from Ref. [36].

Two sensors with 1.2 mm-thick SRRs with ≈22 mm length and a 3 mm gap that is separated from the adjacent transmission line by 0.4 mm are simulated in a full-wave high-frequency simulator (HFSS) [36]. In this simulation, a cylindrical hole inside the substrate is filled with various lossless dielectric materials with $\varepsilon_{r}=2,2.1,2.2,2.3$. The bare sensor resonates around 5GHz when the substrate is chosen to be 0.79 mm-thick Rogers RO5880 with dielectric properties of $\varepsilon_{r}=2.2$ and tan(δ)=0.0009. The transmission response of the sensors ($S_{21}$) is recorded with respect to the variations in the material property, as shown in Figure 15c,d. In the case of external tubing, the tube is flushed on the resonator as shown in Figure 15a (top) and in the embedded channel, a hollow cylinder is emptied out of the substrate and replaced with the tube as shown in Figure 15b (top). The orientation of the tube is under the edge of the SRR as well as the feeding transmission line to improve the sensitivity. Simulation results reveal that the frequency shift in the transmission profile of the sensor with the external channel negligibly changes when the filling material permittivity varies. However, this resonance frequency shift is largely increased in the case of an embedded channel. It can be observed that the resonance frequency shift of the sensor with the external channel is ≈170 MHz/$\Delta \varepsilon_{r}$ while the embedded channel leads to the much higher sensitivity of ≈440 MHz/$\Delta \varepsilon_{r}$ for permittivity variations between 2 and 2.3, leading to an improvement of 250%. The experimental verification of such sensors is also elaborated by measuring common chemicals including IPA ($\varepsilon_{r}^{\prime }=17.9$, $\varepsilon_{r}{}^{\prime \prime }=17.5$), ethanol ($\varepsilon_{r}^{\prime }=24$, $\varepsilon_{r}{}^{\prime \prime }=12$), methanol ($\varepsilon_{r}^{\prime }=30$, $\varepsilon_{r}{}^{\prime \prime }=8$) and acetone ($\varepsilon_{r}^{\prime }=20.7$, $\varepsilon_{r}{}^{\prime \prime }=3.54$). The polytetrafluoroethylene (PTFE) microfluidic tube used in this experiment is with inner/outer diameter of 0.8/1.6 mm, respectively, and 0.4 mm wall thickness. The measured transmission profiles with liquid analytes are shown in Figure 16a,b. Since the comparison is between sensors with the similar bare resonance frequencies, the sensitivity S in this case is defined as $S=\left(f_{res}-f_{0}\right)/\left(\varepsilon_{r}-1\right)$ as computed in detail in Table 3. Frequency shifts in material characterization are engineered to exhibit improvements up to 259% (e.g., IPA) with a change in the sensing configuration, while the sensor architecture as well as the initial operating frequency remain unchanged.

### 2.4. Immobilization of Analytes

**Figure 17:**
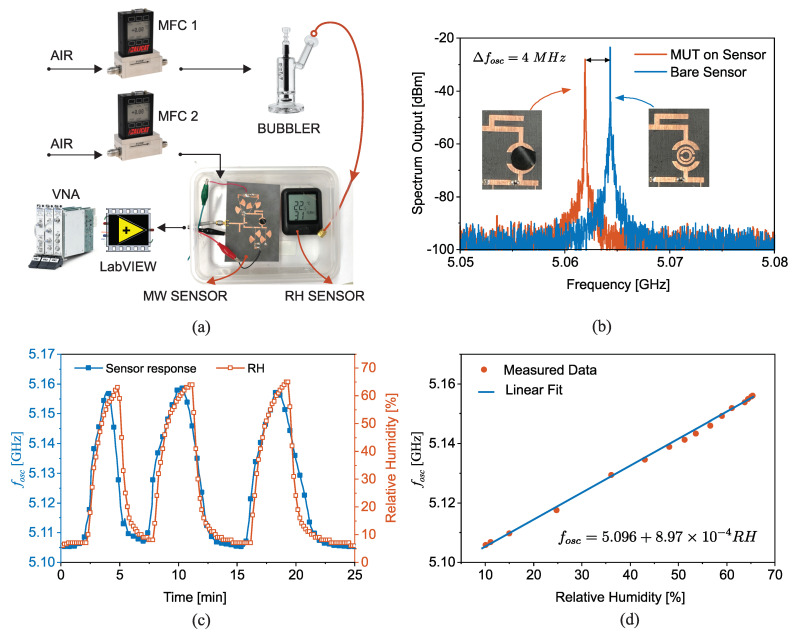
Relative humidity sensor with an immobilizing analyte and sensor response. (**a**) Sensing setup for measuring relative humidity using an oscillator. (**b**) Spectrum output showing the effect of PVA/PANI. (**c**) Cyclic relative humidity measurement and its coherence with the sensor response. (**d**) Calibration curve: oscillation frequency correlation vs. the relative humidity. Reprinted with permission from Ref. [72].

State-of-the-art applications require ultra-high sensitivity for a given MUT. In other words, the sensor response including amplitude, frequency or phase needs to change largely for a tiny variation in the input of the system. This high sensitivity allows for measuring smaller quantities and monitoring the variations with smaller steps. In material characterization applications, enhanced sensitivity enables sensing an infinitesimal change in the dielectric constant such as the ppm levels of a gas in an environment. For one, humidity sensors essentially require ultra-high sensitivities as the effective permittivity in the air changes by 0.012% at the frequency of 5 GHz as a result of the relative humidity (RH) increase from 25% to 75% [69]. This minute change in the RH is difficult to reliably discern with regular microwave sensors due to the low sensitivity of a planar resonator to the permittivity changes of this low value. At the same time, humidity monitoring is pivotal in food processing, semiconductor fabrication, drug manufacturing, etc. [70]. A viable solution for monitoring humidity in these applications is to use an intermediate medium that captures the gaseous medium. Polymer resistance varies with respect to the level of absorbed water content [71]. In addition, large-scale production, synthesis at room temperature, and integration with planar sensors are among the benefits of using intrinsically conducting polymers (ICPs). Once doped by oxidation, ICPs become highly conductive through mobile conjugated p-orbital electrons. Polyaniline (PANI) is an ICP that reacts to the low oxidative potential of water, which also makes it suitable for sensitive humidity as extensively elaborated in [72]. The authors also have used polyvinyl alcohol (PVA) as an adhesive layer between PANI and another substrate while helping water being retained for a longer time. The authors in [72] used PVA/PANI to increase the sensitivity of the sensor for humidity as an immobilizing element which captures the MUT and helps sensitivity enhancement with an increase in the exposure of the resonator to the MUT. A DSRR is used as the sensing element as the tank of a feedback oscillator. The planar resonator is designed at the high frequency of 5GHz to exploit the low self-capacitance of the resonator to achieve high sensitivity. The oscillation condition is met mainly by high gain and phase compensation. A high gain is generated by the amplifier to compensate the resonator loss. Furthermore, proper phasing is required for the positive feedback operation and constructive interference involved in the feedback design. This phase manipulation is implemented using a varactor diode SMV1281 with the capacitance range of 0.1 pF–0.3 pF, which affects both the amplitude and phase of transmission profile. The fabricated sensor is located inside a sealed box that receives humid air through a bubbler. The level of humidity is controlled by a mass flow controller (MFC) that makes the air flow inside the bubbler. To illustrate several cycles of humidity variation, another MFC is used to purge out the existing humid air. The sensor response is continuously recorded by LabView and a commercial logger is utilized to monitor the relative humidity as shown in Figure 17a. A PVA/PANI absorber is placed on the sensor to completely cover the DSRR as shown in Figure 17b. This placement shifts the oscillation frequency down by 4MHz. Then, the sensor performance is examined by increasing the RH of the sealed box from 5%→65% as recorded by the commercial RH logger given in Figure 17c. Corresponding variations in the oscillation frequency are also shown for comparison, that is in great congruence with the environmental variations with minimal delay, and explains that the sensor is in real-time operation and without significant delay. A similar sensor response on the three consecutive cycles reveals that the resonator is repeatable and instantly follows the liquid water removal from the PANI/PVA. This correlation is found to be linear, as given in Figure 17d, where the oscillation frequency is plotted vs. RH and fitted with a linear curve as follows:

(20) $$f_{osc}\;\left[GHz\right]=5.096+8.97\times 10^{-4}RH\;\left(\%\right)$$

This expression translates the RH to the change in the oscillation frequency $f_{osc}$ with a staggering sensitivity of 897 kHz/ΔRH% on the average change of RH:5%→65%. This work represents how an intermediate medium helps aggregate diminutive quantities of MUT to enable sensing them with microwave sensing techniques.

### 2.5. Resonator Pattern Optimization

Nowadays, using computer-aided design (CAD), microwave designers are capable of delivering designs that meet the predefined requirements as long as these requirements are not extreme. For this purpose, they first draw a topology of the design according to the classical methods, and then using optimization techniques, which are embedded in almost all CAD packages, they optimize the sizes, dimensions and other parameters to achieve the required goals. The question which may arise is that of where “it is the best design one can achieve”. Since the optimization is about changing the dimension of the model, not its shape and topology, the performance of the final optimized design highly depends on the initial design topology and so it may not be the best solution. What if there is a way to find the optimum design without having an initial model? If that were the case, the design can be performed in a fully automated, complete-cycle procedure.

Robust methods have been introduced to fully automate and optimize the design of microwave planar devices (fabricated with printed circuit board technology), such as radar cross-section reducer surfaces [73,74,75], high impedance surfaces [76,77], electromagnetic energy harvesting surfaces [78], absorbers [79], decoupling elements between microstrip antennas [80], polarization converters [81,82], and frequency-selective surfaces [83]. In this method, the shape of the artworks (the pattern to be etched into each copper layer of a PCB) is optimized to achieve the best possible performance. This optimization is not about changing the dimensions of a model but rather about its shape. The required optimization procedure is essentially making a decision as to which parts of the pattering area are covered with metal and which parts are not (etched). In particular, this versatile design approach was also applied to the design of high-sensitivity planar sensors [37,84,85]. In microwave sensors based on electrically small planar resonators (ESPRs), the shape of the resonator plays an essential role in the sensitivity. Due to the high-field localization on the ESPR area, in such sensors, the MUT is placed on the resonator area, where there is a strong interaction between the highly localized field with the MUT, which leads to a change in the sensor’s response. The resonator’s shape can affect the field distribution and localization in the sensitive area, and thus the sensitivity. Therefore, the shape of the resonator can be optimized to achieve the highest possible sensitivity.

In [37], a highly sensitive CSRR-based sensor was developed in a complete-cycle topology optimization procedure for the highly accurate characterization of dielectric materials. In this design approach, the sensing area (the CSRR area) was first pixelated. Then, by maximizing the sensitivity as a goal, a binary optimization algorithm was applied to determine whether each pixel was metalized. The outcome of the optimization is a pixelated pattern of the resonator yielding the maximum possible sensitivity. Some details of [37] are provided in the following.

**Figure 18:**
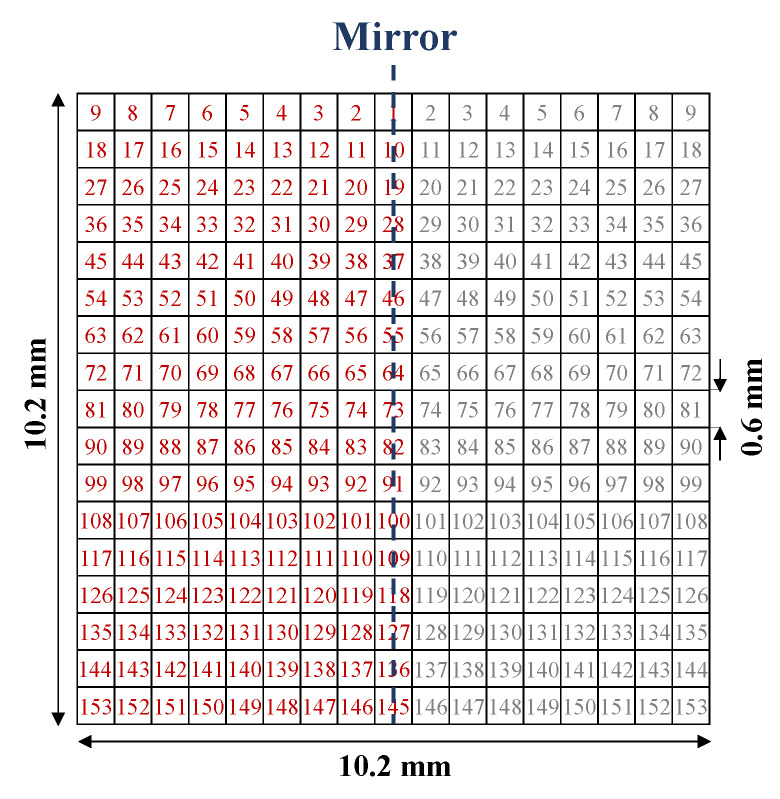
Pixelization of the sensing area. Reprinted with permission from Ref. [37].

By assuming a microstrip line, a square of size 10.2 mm × 10.2 mm in the middle of the ground plane was considered as the sensing area (where the ESPR is etched) and was pixelated into 17×17 pixels, as shown in Figure 18. Then, a binary value of 1 or 0 was assigned to each pixel so that each pixel was represented as a bit whereas having the value of 1 or 0 indicates the presence or absence of metal on the area of the pixel. In such a way, a string of bits represents the shape of the ESPR. Then, the binary version of the particle swarm optimization (PSO), namely BPSO [86,87], was applied to this string (such that each bit is an optimization parameter) to optimize the shape of the resonator for maximum sensitivity.

To reduce the number of independent bits (i.e., the number of optimization parameters) and thus achieve the faster convergence of the optimization algorithm, the design was constrained by enforcing a mirror symmetry with respect to an axis perpendicular to the feed microstrip line. Since setting initial value of the optimization parameters strongly impacts the convergence of every optimization method, to further accelerate the convergence, the initial values of the bits were set such that the initial shape of the resonator was a scaled version of the one used in [88].

To maximize the sensitivity of the sensor to changes in the dielectric constant of the MUT, and since the change in the dielectric constant is sensed by the change in the resonance frequency (when loaded with MUT), the optimization cost function was defined as the inverse of the normalized resonance frequency shift ($\Delta f_{r}^{n}\left(\varepsilon_{r}=10\right)$) due to a material having $\varepsilon_{r}=10$. This choice was because of the characterization of the materials with a dielectric constant ranging between 1 and 10.

**Figure 19:**
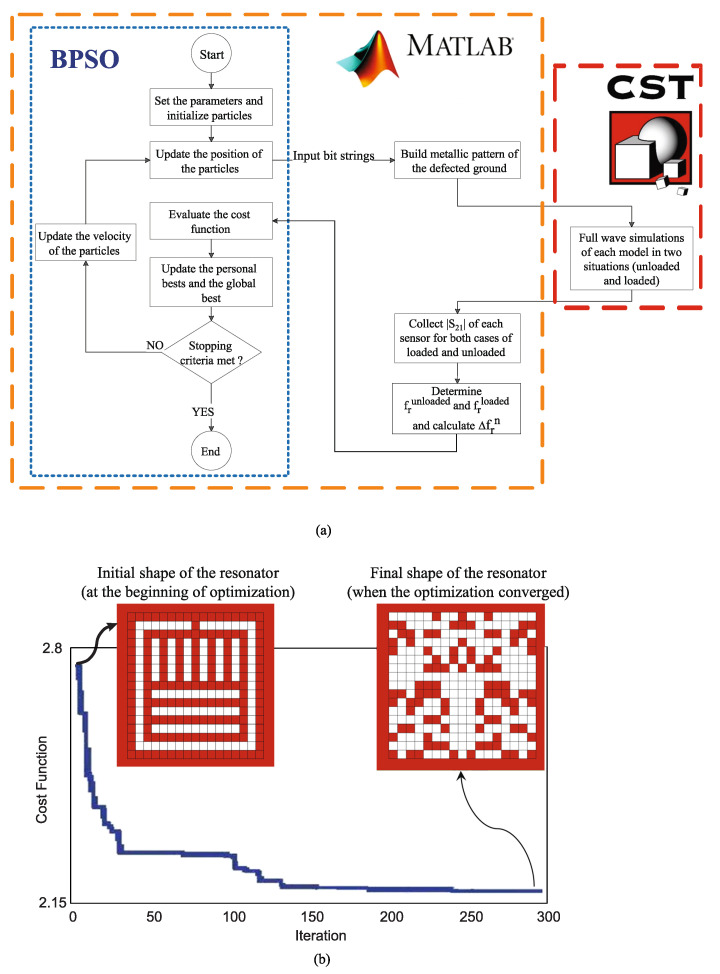
Sensor shape optimization in Matlab coupled with CST: (**a**) flowchart of the design procedure; (**b**) the value of the cost function during the iterations of the optimization algorithm; the left and right insets show the initial and final shapes of the resonator at the beginning and end of the optimization procedure, respectively. Reprinted with permission from Ref. [37].

The process of optimizing the shape of the sensor was performed by writing a code in MATLAB and linking it to the CST as an electromagnetic full-wave simulator. The diagram of this process, including the flowchart of the BPSO algorithm and the relationship between MATLAB and CST, is shown in Figure 19a.

In this process, after setting the initial parameters and generating binary strings, each string is converted to a pattern which can be simulated in CST. In CST, each model (sensor) is simulated when it is unloaded and when loaded with a sample having $\varepsilon_{r}=10$. Then, the results of the full-wave EM simulations are fed back to MATLAB. Using the received transmission coefficients, the normalized resonance frequency shift of the loaded sensor (with respect to the unloaded state) is determined and the cost function is calculated. Figure 19b shows the values of the cost function during the iterations of the algorithm indicating the decrease in the cost function from 2.78 (for the initial shape of the sensor) to 2.17 (for the final shape), or equivalently $\Delta f_{r}^{n}\left({\varepsilon_{r}}^{\prime \prime }=10\right)$ was increased from 0.36 to 0.46. The pattern of the optimized sensor is shown in the inset of Figure 19b.

**Figure 20:**
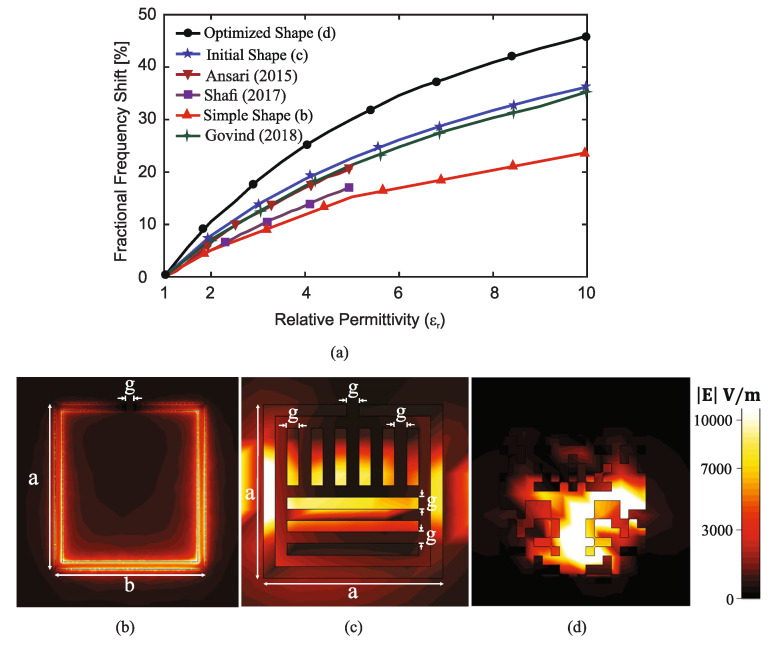
Optimized sensor response with field intensity. (**a**) Comparison between the sensitivity of the proposed shape-optimized sensor with that of the earlier works of Ansari (2015) [89], Shafi (2017) [90], and Govind (2018) [91]. (**b**–**d**): the electric field intensity distribution on a simple CSRR resonator, the resonator which was considered as the initial state of the optimization and the shape-optimized resonator, respectively. Reprinted with permission from Ref. [37].

By gradually increasing the relative permittivity of the MUT from 1 to 10 in the simulations, in Figure 20a, the normalized resonant frequency shift of the optimized sensor was compared with that of a simple CSRR sensor, the modified CSRR sensor which was considered as the initial state of the optimization, and those introduced in three other works (notice that the reference numbers in Figure 20 shows the references of [37]). It is evident that for every dielectric constant of MUT, the optimized sensor using pixelization and shape optimization exhibits the highest normalized resonant frequency shift. In particular, for dielectric constants of 2 and 10, this sensor shows normalized resonant frequency shifts of 10% and 46%. To understand the underlying mechanism behind this improvement, Figure 20b–d show a comparison between the field intensity distributions on the simple CSRR, the resonator which was considered as the initial state of the optimization and the proposed shape-optimized resonator. From this comparison, it can be concluded that the sensitivity improvement in the pixelated sensor is due to (1) a stronger field on the sensing area such that the maximum intensity of the electric field is three times higher than that in Figure 20c, and (2) a larger effective sensing area wherein the electric field has a strong intensity. These two features provide broader and more effective coupling (interaction) between the sensor and the MUT.

**Figure 21:**
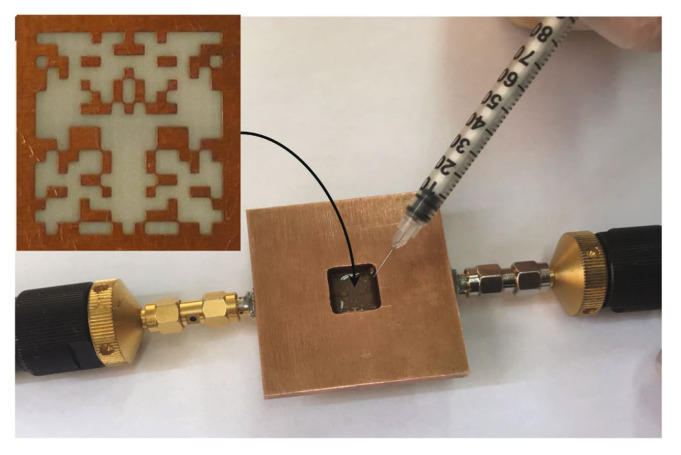
Measurement of a dielectric liquid material using the fabricated sensor and a VNA; the inset shows a zoomed-in view of the pixelated pattern of the resonator which was etched on the ground plane of the microstrip line.

**Figure 22:**
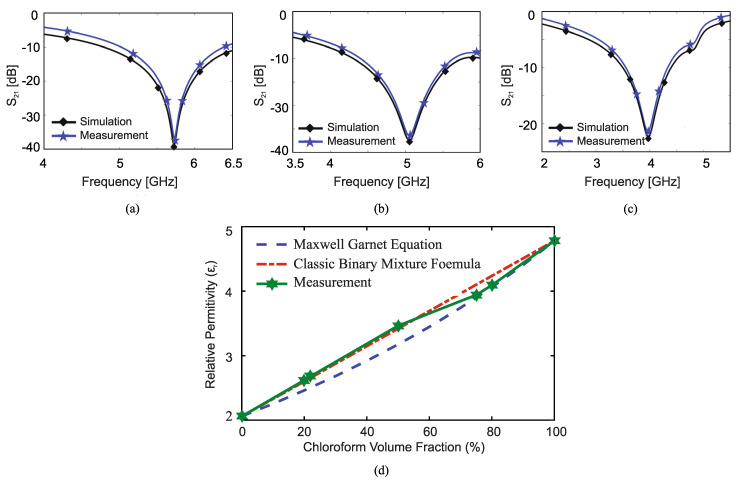
Comparisons between the simulated and measured $\left|S_{21}\right|$ of the sensor when it is unloaded (**a**) and is loaded with cyclohexane (**b**) and chloroform (**c**); (**d**) the dielectric constant of the mixture of chloroform and cyclohexane as a function of the volume fraction of chloroform. Reprinted with permission from Ref. [37].

Experimental validation was also presented in [37]. The optimized sensor was fabricated by etching the ground plane of a 50 Ω microstrip line as shown in Figure 21. A copper plate with a hole in the middle was placed on the ground plane of the board. The hole is slightly larger than the sensing area, thus effectively creating a small container that includes the sensor’s area in the bottom and an open top for inputting or pouring in the MUT. Two different tests were performed. In the first, the transmission coefficient ($\left|S_{21}\right|$) of the sensor when it was unloaded and was loaded with cyclohexane ($C_{6}H_{12}$) and chloroform (${\mathrm{CHCl}}_{3}$) having a known relative permittivity of 2.02 and 4.81 was measured. The measured and simulated transmission coefficients are seen in Figure 22a–c, showing strong agreement between the simulations and experiments.

In the second set of experiments, the fabricated sensor was used to extract the dielectric constant of mixtures of chloroform and cyclohexane with different volume ratios. Each sample was tested with the optimized sensor and the transmission coefficient was measured and the normalized resonance frequency shift was obtained. Using the relation between the normalized frequency shift and the dielectric constant of the MUT (shown in Figure 20), the relative permittivity of each mixture was obtained and compared with those approximated by the classic binary mixture and the Maxwell–Garnett formulas, as shown in Figure 22d. Good agreement between the results is observed.

**Figure 23:**
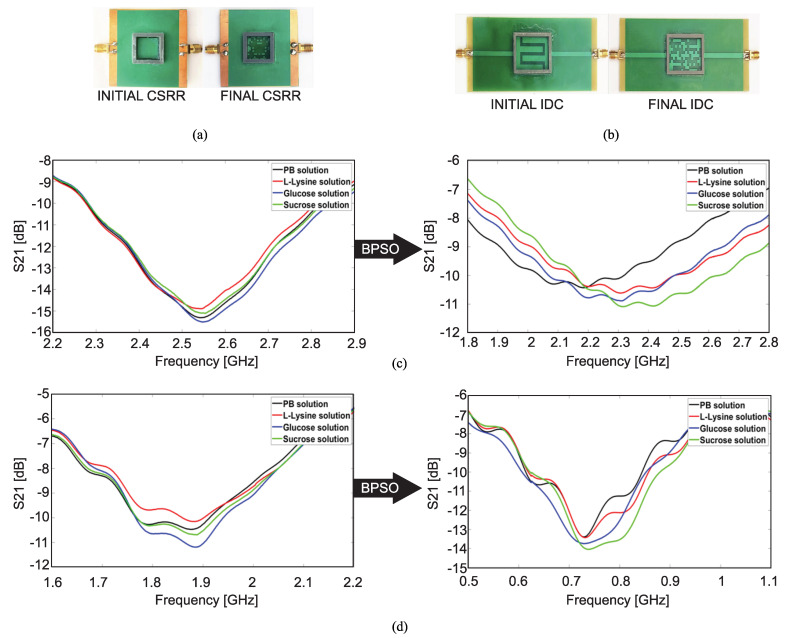
The initial and optimized (**a**) CSRR and (**b**) IDC sensors. The response of the initial and optimized CSRR (**c**) and IDC (**d**) sensors. Reprinted with permission from Ref. [84].

In a similar work [84], the same approach, i.e., the pixelization of the sensing area (resonator area) and applying a binary optimization algorithm was used to design CSRR and interdigitated capacitor (IDC)-based sensors. While in the CSRR sensor, a resonator is etched on the ground plane of a microstrip line, in the IDC sensor, the resonator (which consists of inductive inter-digitated fingers with capacitive gaps between them) is placed between two sections of a microstrip line. Figure 23a,b show the fabricated CSRR and IDC sensors before (the design which was given to the BPSO algorithm as an initial design) and after resonator shape optimization.

The sensors in this work were designed for bio-sensing applications, where most of the samples are dissolved in respective solvents. Hence, the normalized frequency shift was calculated with respect to a solvent which was a solution of 34 g of phosphate buffer (PB) in one liter of deionized water. This PB solution was used as the solvent of other samples: L-Lysine, glucose and sucrose. Figure 23 shows the measured responses of the CSRR (a) and IDC (b) sensors before and after optimization (transmission and reflection coefficients in the cases of CSRR and IDC sensors, respectively). It is seen that the normalized frequency shifts after the shape optimization of the resonator increased in both cases, which is identical to sensitivity improvement.

In the two aforementioned works [37,84], the optimum shape of the resonator was obtained by applying a binary swarm optimization. Although the swarm intelligence algorithms are easy to implement, for large-scale and fine-grained optimization problems, the demands of the number of dimensions and the population of the solution are high, resulting in a slow modeling speed and high iteration number. To address this problem, in [85], deep reinforcement learning was applied to optimize the shape of the resonator etched on the ground plane of a microstrip line in a microwave microfluidic sensor.

**Figure 24:**
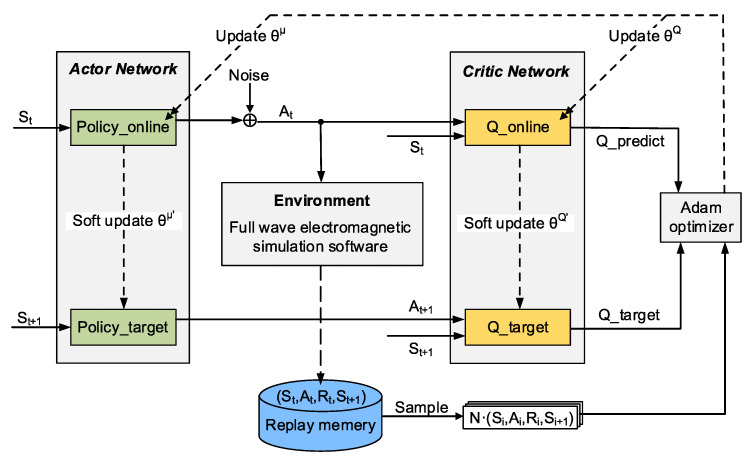
Framework of the DDPG. Reprinted/adapted with permission from Ref. in [85].

**Figure 25:**
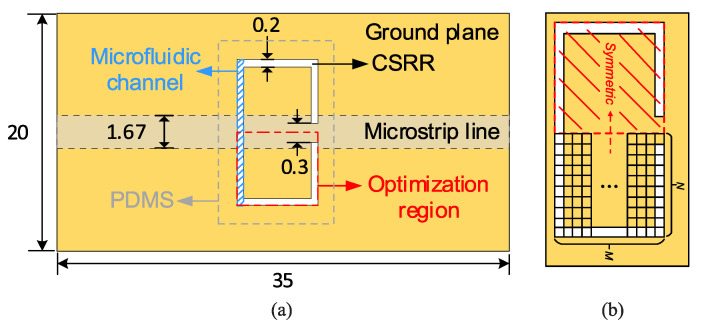
Design flow of the CSRR pixelation: (**a**) initial design of the microfluidic sensor based on a classical CSRR (all the dimensions are in mm); (**b**) pixelated CSRR structure. Reprinted with permission from Ref. [85].

A deep deterministic policy gradient (DDPG) [92] framework, as shown in Figure 24, including actor and critic networks (both are neural networks) and a full-wave electromagnetic simulator (HFSS), was employed as an agent for learning an optimization strategy. In this strategy, as shown in Figure 25, by considering one-axis symmetry for the resonator, half of the resonator area was pixelated, and a bit of 0 or 1 corresponding etched or unetched was assigned to each pixel. Therefore, a string of bits shows the state space. The agent would determine a series of structural adjustment actions according to the current state and the learned knowledge. The agent would receive a reward according to the state and action. As the goal of the optimization was to find out the resonator structure with the largest relative resonant frequency shift (i.e., the optimal sensitivity) when loaded with the samples through a series of action adjustments, the main reward factor is the relative resonant frequency shift of the loaded sensor. Meanwhile, the normalization of the microfluidic channel was also considered as the channel which is bonded onto the resonator structure. The incorrect actions that make the microfluidic channel difficult to fabricate or affect the liquid flow were defined, and a penalty was considered for them in the reward function.

The optimization procedure of the DDPG agent can be described as follows. The agents would gradually learn in the process of continuous interaction with the simulation environment. As such, the agent chooses the actions to adjust the resonator structure, the model script generated by the program is constructed in the electromagnetic simulation software and the simulated results are used as the output to evaluate the performance of the resonator structure. Then, the program provides the reward value and goes to the next episode until the actor and critic networks eventually converge. The optimal solution could be obtained and output as the optimal resonator structure of the microwave microfluidic sensor.

A classical complementary split-ring resonator structure (shown in Figure 25) was used as an initial structure and optimized in two ways, i.e., with and without a fixed liquid volume consumption. In the former optimization, the goal was to maximize the relative resonant frequency shift when the volume of the fluid sample under test was fixed to 2.4 μL. However, in the latter, the goal was to maximize the resonance frequency shift and minimize the sample volume at the same time. As a result, a liquid volume of 0.8 μL was needed for the optimized sensor in the second way.

**Figure 26:**
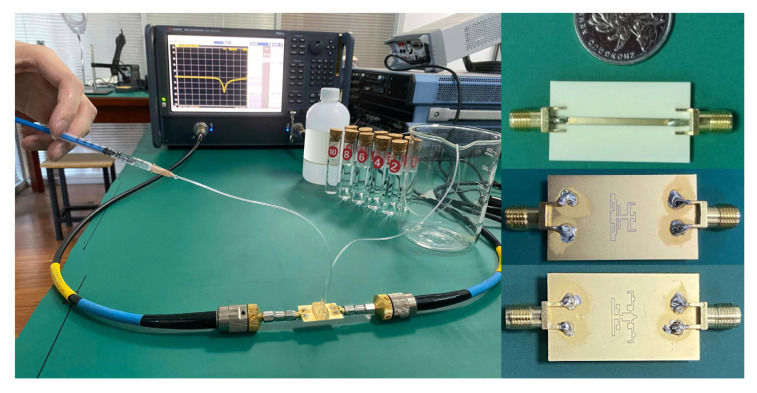
Photographs of the experimental setup and the optimized sensors. The right, middle is the sensor optimized to minimize the volume of the liquid sample and the right, bottom is that optimized with a fixed volume of sample under test. Reprinted with permission from Ref. [85].

**Figure 27:**
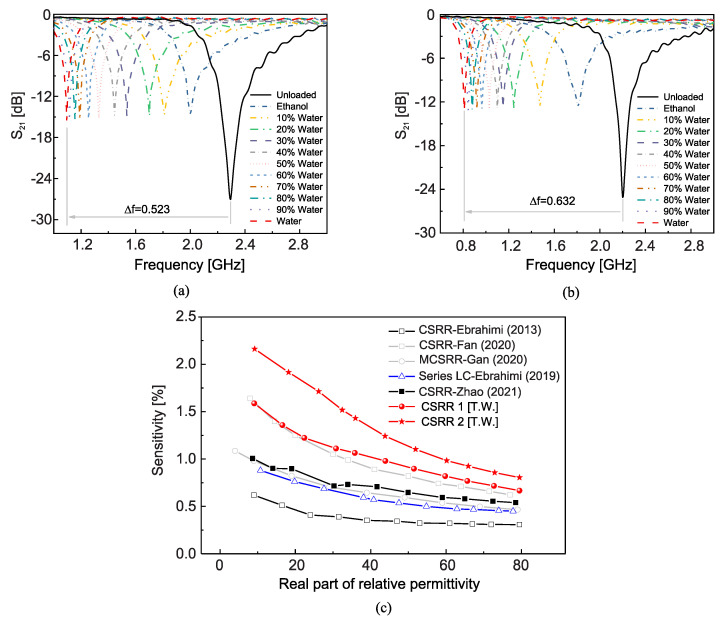
The transmission response of the optimized sensors: (**a**) the one optimized to minimize the volume of the liquid sample and (**b**) the one optimized with a fixed volume of sample under test. (**c**) Comparison between the sensitivity of the sensors fabricated in [85] and some other CSRR-based sensors as follows: Ebrahimi (2013) [65], Fan (2020) [93], Gan (2020) [94], Ebrahimi (2019) [21], Zhao (2021) [95]. Reprinted with permission from Ref. [85].

As shown in Figure 26, the optimized sensors were fabricated and tested with water–ethanol solutions in different ratios as the samples under test. The transmission responses of the sensors in their unloaded and loaded states are given in Figure 27a,b.

By defining the sensitivity as

(21) $$S=\frac{f_{\mathrm{unloaded}}-f_{\varepsilon_{r}}}{f_{\mathrm{unloaded}}}\;\;\cdot \;\;\frac{1}{\varepsilon_{r}-1}$$

Figure 27c compares the sensitivity of the sensors proposed in [85] with that of some other CSRR-based sensors (the reference numbers are for [85]). Considerable improvement of the sensitivity is observed.

### 2.6. Incorporating Phase Variation

**Figure 28:**
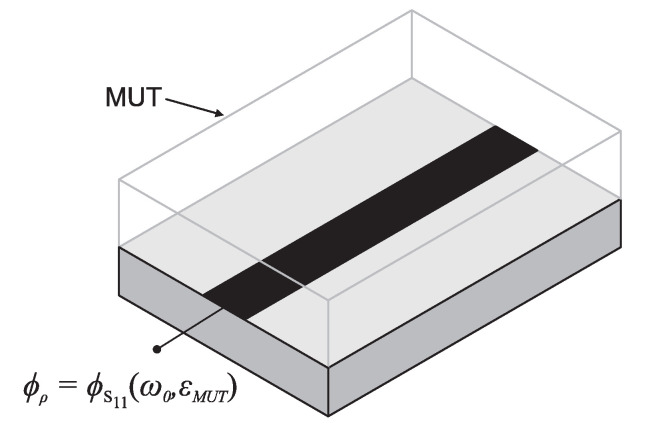
Sketch, in perspective view, of an open-ended sensing line.

In this subsection, a strategy to significantly enhance the sensitivity in phase-variation microwave sensors is reviewed. Such a technique is applied to one-port reflective-mode sensors, where the sensing element is a resonant element, either distributed or semi-lumped, and it consists of cascading to a sensing element such as a set of quarter-wavelength transmission line sections with alternating high and low impedance [96,97,98,99]. The considered phase-variation sensors operate at a single frequency, and their canonical output variable is the phase of the reflection coefficient, whereas the natural input variable is the dielectric constant of the material under test (MUT), which should be in contact with the sensing element. The simplest implementations of phase-variation sensors consist in a one-port (reflective-mode) [39,96,100,101,102,103] or a two-port (transmission-mode) [38,104,105,106] transmission line, the sensing element. When a material is in contact with the line, the effective dielectric constant of such a line is modified, and consequently, the phase velocity and the characteristic impedance are also altered. The result is a variation of the electrical length of the line and, ultimately, a variation in the phase of the reflection (reflective-mode) or transmission (transmission-mode) coefficient of the line is also generated. The electrical length of the line is given by

(22) $$\phi =\beta l=\frac{\omega_{0}}{v_{p}}l$$

where β is the phase constant of the line and l its length, $\omega_{0}$ and $v_{p}$ being the angular frequency and the phase velocity, respectively. Although ϕ is not the typical output variable (it only coincides with the phase of the transmission coefficient of the line in matched lines), from (22), it can be easily deduced that the variation of the phase (electrical length) of the line with the dielectric constant of the MUT, the input variable, will increase by increasing either the frequency or the length of the line, or both. Thus, it can be concluded that the sensitivity (or derivative of the output variable, the phase of the reflection or transmission coefficient, with the dielectric constant of the MUT) increases with the operating frequency or with the line length. Indeed, highly sensitive sensors based on long (typically meandered) sensing lines have been reported [105,106]. However, this obvious strategy has the penalty of large sensing regions, provided that a high sensitivity is required. Increasing the operating frequency, the other canonical sensitivity enhancement strategy in phase-variation sensors, is not always applicable (sometimes, the operating frequency is dictated by external factors), or, if applicable, it may represent an increase in the cost of the associated electronics for signal generation and processing in a real scenario. To enhance the sensitivity in one-port phase-variation sensors, a novel strategy was pointed out in [96]. In the simplest form, the sensor consists of an open-ended line with impedance $Z_{s}$ and electrical length $\phi_{s}$ (at the operating frequency) when the line is loaded with the reference material (e.g., air), as can be seen in Figure 28.

The output variable is the phase of the reflection coefficient, $\phi_{s}$, which varies when a material under test (MUT) is in contact with the line. To obtain the dependence of such an output variable with the parameters of the line, it is first necessary to infer the impedance seen from the input port, given by [52,96]

(23) $$Z_{in}=-jZ_{s}cot\phi_{s}$$

The reflection coefficient is thus

(24) $$\rho =\frac{Z_{in}-Z_{0}}{Z_{in}+Z_{0}}=\frac{+Z_{0}+jZ_{s}cot\phi_{s}}{-Z_{0}+jZ_{s}cot\phi_{s}}$$

and the phase of the reflection coefficient is therefore

(25) $$\phi_{\rho }=2arctan\left(\frac{Z_{s}cot\phi_{s}}{Z_{0}}\right)+\pi =2arctan\left(\frac{Z_{s}tan\phi_{s}}{Z_{s}}\right)$$

The sensitivity can be expressed as:

(26) $$S=\frac{d\phi_{s}}{d\varepsilon_{\mathrm{MUT}}}=\frac{d\phi_{\rho }}{d\phi_{s}}\;\cdot \;\frac{d\phi_{s}}{d\varepsilon_{\mathrm{MUT}}}+\frac{d\phi_{\rho }}{dZ_{s}}\;\cdot \;\frac{dZ_{s}}{d\varepsilon_{\mathrm{MUT}}}$$

where the different derivatives appearing in (26) are given by

(27) $$\frac{d\phi_{\rho }}{d\phi_{s}}=-\frac{2}{\frac{Z_{0}}{Z_{s}}sin^{2}\phi_{s}+\frac{Z_{s}}{Z_{0}}cos^{2}\phi_{s}},$$

(28) $$\frac{d\phi_{\rho }}{dZ_{s}}=\frac{2}{1+{\left(\frac{Z_{s}}{Z_{0}}\right)}^{2}cot\phi_{s}}\;\cdot \;\frac{cot^{2}\phi_{s}}{Z_{0}}$$

(29) $$\frac{d\phi_{s}}{d\varepsilon_{\mathrm{MUT}}}=\frac{\phi_{s}}{4\varepsilon_{e}ff}\left(1-F\right)$$

(30) $$\frac{dZ_{s}}{d\varepsilon_{\mathrm{MUT}}}=\frac{Z_{s}}{4\varepsilon_{e}ff}\left(1-F\right)$$

The derivatives (29,30) correspond to a microstrip sensing line, $\varepsilon_{eff}$ and *F* being the effective dielectric constant and the form factor as defined in [52,107], respectively, (for CPWs, the same expressions apply, but forcing the geometry factor to be null, *F* = 0). Introducing (27) in (26), the sensitivity can be expressed as

(31) $$S=-\frac{Z_{s}\left(1-F\right)}{2Z_{0}\varepsilon_{eff}}\;\cdot \;\frac{\phi_{s}sin^{-2}\phi_{s}+cot\phi_{s}}{1+{\left(\frac{Z_{s}}{Z_{0}}\right)}^{2}cot^{2}\phi_{s}}$$

As demonstrated in [52,96], the sensitivity in the limit of small perturbations is optimized by choosing $\phi_{s}=\left(2n+1\right)\;\cdot \;\pi /2$ and $Z_{s}$ as high as possible (as compared to $Z_{0}$), or alternatively, by forcing $\phi_{s}=n\;\cdot \;\pi$ and $Z_{s}$ as low as possible. In the former case, the sensitivity is given by

(32) $$S=-\frac{Z_{s}\left(1-F\right)}{2Z_{0}\varepsilon_{eff}}\phi_{s}$$

In the second case ($\phi_{s}=n\;\cdot \;\pi$ and $Z_{s}$ low), the sensitivity is calculated as

(33) $$S=-\frac{Z_{0}\left(1-F\right)}{2Z_{s}\varepsilon_{eff}}\phi_{s}$$

**Figure 29:**
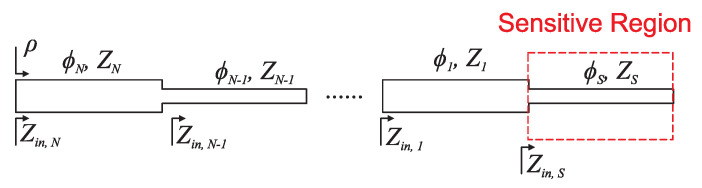
Schematic of the phase-variation sensor based on a sensing line and a step-impedance transmission line (with quarter-wavelength line sections). Reprinted with permission from Ref. [96].

According to (32) and (33), it is clear that the sensitivity is enhanced by considering the sensing lines with a high index *n*, that is, many (odd) multiples of a quarter-wavelength, or many multiples of a half-wavelength, depending on the ratio between $Z_{s}$ and $Z_{0}$. However, with this strategy, the length of the sensing line may be excessive. In [96], it was demonstrated that by simply alternating between cascading high- and low-impedance quarter-wavelength transmission line sections, the sensitivity can be substantially enhanced (see Figure 29).

For $\phi_{s}=\left(2n+1\right)\;\cdot \;\pi /2$ and high-impedance sensing lines, the $90^{\circ }$ line section adjacent to the sensing line must exhibit a low characteristic impedance, whereas for $\phi_{s}=n\;\cdot \;\pi$ and low impedance sensing lines, the characteristic impedance of the quarter-wavelength line section cascaded to it must be high. If the impedances of such high/low $90^{\circ }$ lines are designated with the variable $Z_{i}$, where *i* indicates the position of the line section (with i=1 corresponding to the line adjacent to the sensing line), the sensitivity can be expressed according to four cases, depending on whether the total number of quarter-wavelength high/low-impedance line sections, *N*, is odd or even, that is [52,96]:

Case $A^{\prime }:\phi_{s}=\left(2n+1\right)\;\cdot \;\pi /2$ and *N* odd.

(34) $$S=-\frac{Z_{s}Z_{0}}{\prod_{i=1}^{N}Z_{i}^{2.{\left(-1\right)}^{i+N}}}\;\cdot \;\frac{\phi_{s}}{2\varepsilon_{eff}}\left(1-F\right)$$

Case $B^{\prime }:\phi_{s}=n\;\cdot \;\pi$ and *N* odd.

(35) $$S=-\frac{\prod_{i=1}^{N}Z_{i}^{2.{\left(-1\right)}^{i+N}}}{Z_{s}Z_{0}}\;\cdot \;\frac{\phi_{s}}{2\varepsilon_{eff}}\left(1-F\right)$$

Case $C^{\prime }:\phi_{s}=\left(2n+1\right)\;\cdot \;\pi /2$ and *N* even.

(36) $$S=-\frac{\prod_{i=1}^{N}Z_{i}^{2.{\left(-1\right)}^{i+N}}}{Z_{0}}\;\cdot \;\frac{\phi_{s}}{2\varepsilon_{eff}}\left(1-F\right)$$

Case $D^{\prime }:=\phi_{s}=n\;\cdot \;\pi$ and *N* even.

(37) $$S=-\frac{Z_{0}}{\prod_{i=1}^{N}Z_{i}^{2.{\left(-1\right)}^{i+N}}}\;\cdot \;\frac{\phi_{s}}{2\varepsilon_{eff}}\left(1-F\right)$$

**Figure 30:**
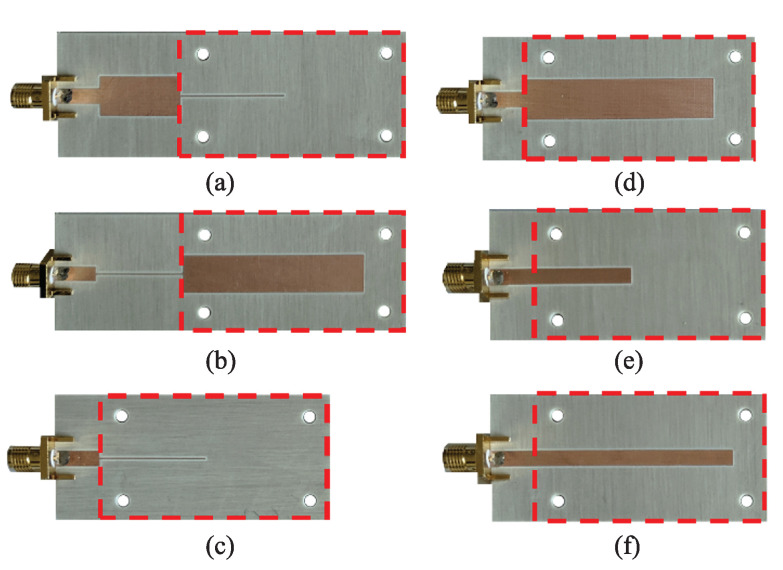
Schematic of the phase-variation sensor based on a sensing line and a step-impedance transmission line (with quarter-wavelength line sections). (**a**) Sensor with step-impedance discontinuity where $Z<Z_{0}<Z_{s}$. (**b**) Sensor with step-impedance discontinuity where $Z>Z_{0}>Z_{s}$. (**c**) Sensor with uniform mismatched sensing line where $Z_{s}>Z_{0}$. (**d**) Sensor with uniform mismatched sensing line where $Z_{s}<Z_{0}$. (**e**) Sensor based on a $90^{\circ }$ uniform 50-Ω sensing line. (**f**) Sensor based on a 180${}^{\circ }$ uniform 50-Ω sensing line. Reprinted with permission from Ref. [96].

**Figure 31:**
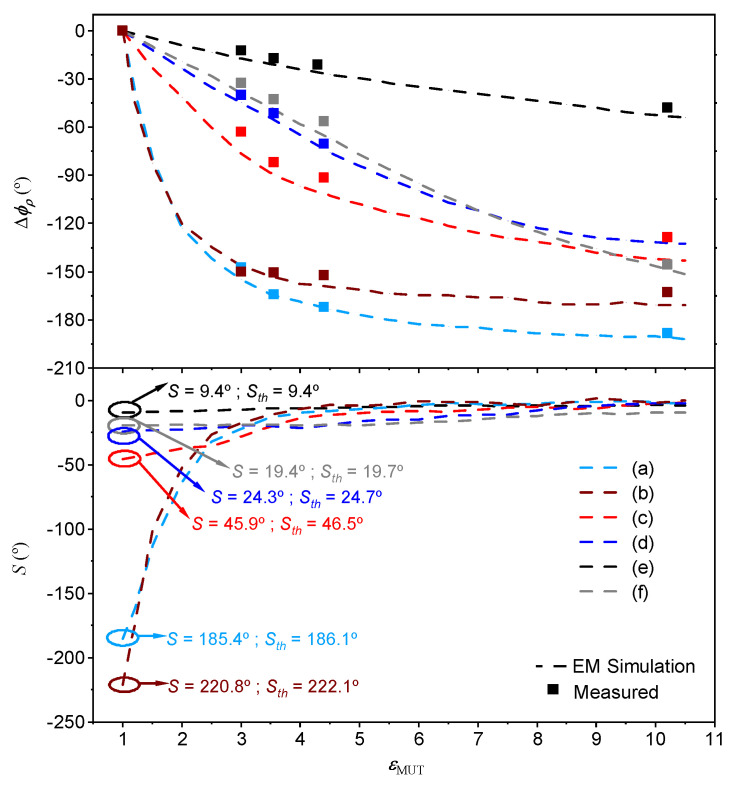
Measured and simulated phase of the reflection coefficient for the sensors of Figure 30, and simulated sensitivity: (**a**) sensor with step-impedance discontinuity and $Z_{1}<Z_{0}<Z_{s}$; (**b**) sensor with step-impedance discontinuity and $Z_{1}>Z_{0}>Z_{s}$; (**c**) sensor with uniform mismatched sensing line and $Z_{s}>Z_{0}$; (**d**) sensor with uniform mismatched sensing line and $Z_{s}<Z_{0}$; (**e**) sensor based on a $90^{\circ }$ uniform 50-Ω sensing line; and (**f**), sensor based on a $180^{\circ }$ uniform 50-Ω sensing line. The measured dielectric loads are 3 mm slabs of uncoated PLA ($\varepsilon_{\mathrm{MUT}}=3$), Rogers RO4003C ($\varepsilon_{\mathrm{MUT}}=3.55$), FR4 ($\varepsilon_{\mathrm{MUT}}=4.4$) and Rogers RO3010 ($\varepsilon_{\mathrm{MUT}}=0.2$) substrates. The sensitivities in the limit of small perturbations are given in absolute values. Reprinted with permission from Ref. [96].

To illustrate the potential of this approach, based on a $90^{\circ }$ or a $180^{\circ }$ sensing line and a step impedance configuration, Figure 30 shows the photograph of different sensors, whereas Figure 4 depicts the phase variation of the reflection coefficient with the dielectric constant of the MUT and the sensitivity [96]. Note that the considered reference MUT is air, which means that the electrical length of the sensing lines (either $\phi_{s}=90^{\circ }$ or $\phi_{s}=180^{\circ }$, depending on the case) is for the bare sensing line at the considered operating frequency (2GHz). The impedance $Z_{s}$ of this line is also for the bare sensing line. The sensitivities in the limit of small perturbations are indicated in Figure 31 for each sensor, and it can be seen that they coincide to a good approximation with the theoretical sensitivities ($S_{th}$ in the figure), as inferred from expressions (34)–(37). For sensors (a) and (b), the sensitivities in the limit of small perturbations are very high, but it should be mentioned that such high sensitivities are achieved at the expense of degradation in the sensor linearity. Thus, these highly sensitive phase-variation sensors are of special interest in applications where the measurement of small perturbations in the input variable with regard to the reference one should be measured. Although these sensors are canonically permittivity sensors, the input variable can be any other variable related to the permittivity, for example, the level of concentration of solute in diluted solutions, or even other variables such as temperature or humidity, which can modify the dielectric constant of certain materials [52]. Let us also mention that, with these high sensitivities, these sensors are very interesting for the detection of tiny defects in samples, which typically manifest as variations in the effective dielectric constant of the sample.

In the reported implementations of Figure 30, the sensing elements are $90^{\circ }$ or $180^{\circ }$ open-ended lines, i.e., distributed resonators. However, such distributed resonators can be replaced with electrically small semi-lumped resonators, as demonstrated in [39], where a reflective-mode phase-variation sensor based on an open complementary split-ring resonator (OCSRR) terminating a step impedance CPW structure was reported. It has also been demonstrated that this type of phase-variation sensor is useful for the measurement of short-range displacements [108] and can be also useful for the measurement of liquid levels.

### 2.7. Exploiting the Coupled-Line Sections

**Figure 32:**
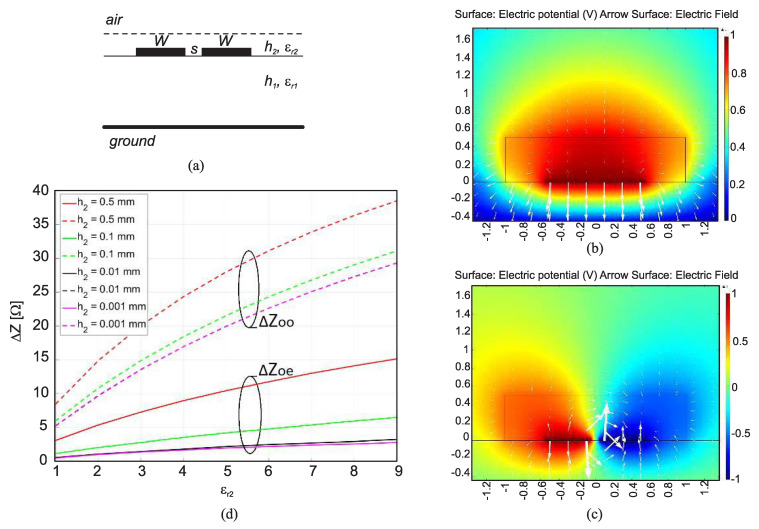
Microstrip line with even/odd mode fields and characteristic impedance: (**a**) a coupled line microstrip section loaded by a dielectric sample. The electric voltage (colors) and field (vectors) distribution for the even (**b**) and odd (**c**) modes. (**d**) Change of the even and odd mode characteristic impedances. Reprinted with permission from Ref. [40].

It has been shown that coupled line sections are applicable for high-sensitivity dielectric sample detection. The idea of using a coupled-line section as a dielectric constant sensor was first proposed by Piekarz et al. in 2015 [40] and then developed in their subsequent works [109,110]. The main thought behind their studies was to exploit the odd-mode characteristic impedance of a coupled line section. This is because putting a dielectric sample on the top of a coupled-line section significantly influences its odd-mode characteristic impedance, which is related to the mutual capacitance between coupled strips, not merely the capacitance between the strips and the ground plane as in a single section microstrip line. Figure 32a shows a microstrip coupled-line section covered with a dielectric sample. Figure 32b,c depict the electric voltage (colors) and field (vectors) distribution in the cross-section for the even and odd modes excitation, respectively, (i.e., when the two lines are excited in phase and out of phase with the same amplitude). It is clear that the field is within the substrate for the even mode excitation, while in the case of odd mode excitation, the field is partially within the dielectric sample above the line. Therefore, the coupled lines’ odd-mode impedance exhibits a much higher sensitivity on the dielectric cover than the even-mode impedance, as observed in Figure 32d different thicknesses and the permittivities of the covering material. Moreover, the sample can be as narrow as the distance between the coupled lines due to the field distribution, shown in Figure 32c.

**Figure 33:**
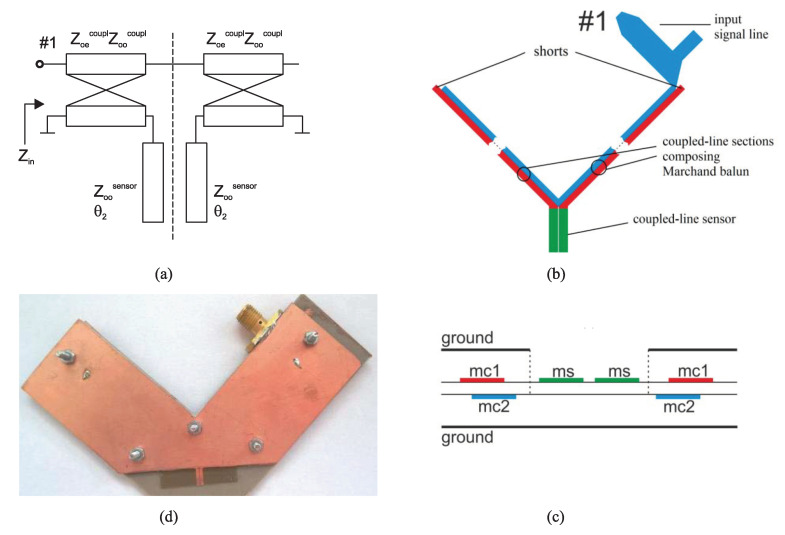
The measurement setup composed of a Marchand balun and an open-ended coupled-line sensor: (**a**) schematic and (**b**) layout and (**c**) cross-sectional views and (**d**) fabricated setup. Reprinted with permission from Ref. [40].

In their first work [40], as shown in Figure 33, Piekarz et al. used a Marchand balun to excite an open-ended coupled-line sensor out of phase in a wide-band. The Marchand balun was designed based on broadside-coupled stripline, as can be seen in Figure 33b. The measurement setup, including the Marchand balun and the open-ended coupled-line section (sensor), is shown in Figure 33c. The material under test is placed on the sensor and the characterization mechanism is as follows. First, the reflection coefficient at port 1 (see Figure 33a) is measured, and its input impedance is obtained. Then, using analytic relations, the input impedance of the sensor section (open-ended coupled-line section), namely the load impedance, is calculated. Notice that since the balun on the differential ports excites the coupled-line section in its odd mode, the load impedance only depends on the odd mode characteristic impedance and the electrical length of this section. Subsequently, after some mathematical manipulations, the odd-mode effective dielectric constant can be calculated using the obtained load impedance and the odd-mode per-unit-length capacitance of the unloaded coupled-line sensor determined during the calibration process. It should be noted that this procedure does not give the dielectric constant of the MUT but the odd-mode effective dielectric constant of the coupled line section, which is between the dielectric constants of the MUT and substrate.

**Figure 34:**
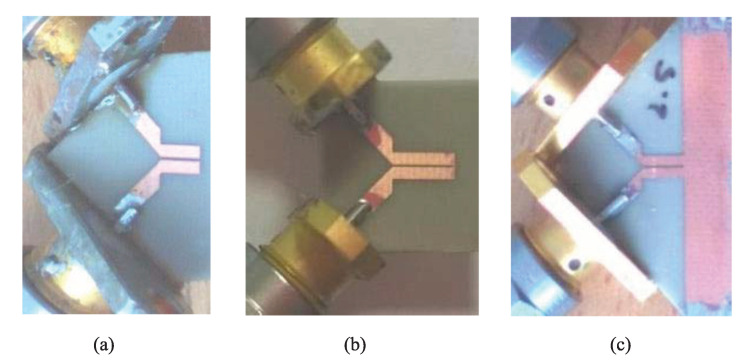
The fabricated coupled line sensors: (**a**) open-ended; (**b**) short ended with small connecting segment; and (**c**) short-ended with grounded connecting pad. Reprinted with permission from Ref. [109].

In their second work, the same authors removed the need for Marchand balun and performed two-port single-ended S-parameter measurements of the coupled line section. The differential input impedance of the sensor (i.e., the input impedance of the section for the odd mode excitation) was calculated from the two-port S-parameters, then, following a procedure similar to their first work, the odd-mode effective permittivity was obtained. As an advancement over their first work, the odd-mode effective permittivity of the coupled line section was finally converted to the complex permittivity of MUT using an electromagnetic (EM) simulation tool in an iterative procedure. In addition, in the newer work, not only an open-ended coupled line section but also short-ended sections where the two coupled lines were connected with a small metallic segment or a large grounded pad were theoretically and experimentally investigated. The three fabricated sensors are depicted in Figure 34. It has been shown that the short-ended sensors have better accuracy for high frequencies (still in the low gigahertz regime), while the open-ended section is suitable for characterizing samples, which feature relatively significant permittivity change for the lower frequencies. Moreover, the calibration process is easier for the sensor shown in Figure 34c, i.e., when the two coupled lines are connected using a grounded pad.

**Figure 35:**
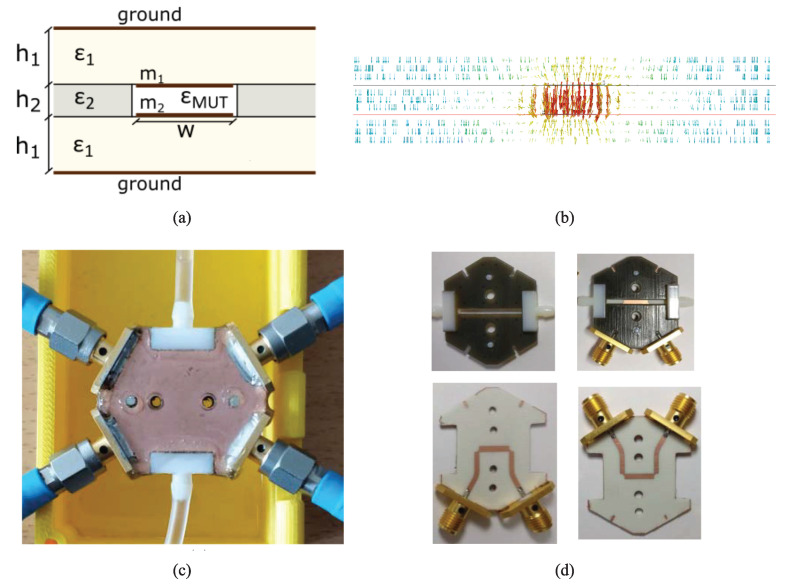
Broadside-coupled line sensor: (**a**) cross-sectional view; (**b**) electric field distribution within the cross section under a differential excitation; (**c**) the microwave microfluidic setup; and (**d**) photographs of the sensor’s components and assembly. Reprinted with permission from Ref. [110].

In the most recent work of the same group [110], as shown in Figure 35a, a four-port broadside-coupled line section based on a stripline was utilized to determine the dielectric constant of liquid samples. A microfluidic channel was realized using 3D printing technology in between the two aligned coupled strips. Figure 35b shows the electric field distribution within the cross-section of the broadside-coupled-line section under a differential excitation. As can be seen, unlike the field distribution in an edge-coupled line sensor (shown in Figure 35c) in the broadside-coupled-line sensor, the field is confined within the sample volume (between the two strips); hence, a higher sensitivity. Moreover, in the broadside-coupled-line sensor, the odd-mode effective permittivity is identical to the permittivity of the MUT. Therefore, the need for a computer simulation-based procedure to convert the effective permittivity to the permittivity of the MUT was removed. Experimental validation was presented by fabricating the sensor and performing four-port measurements for various liquid samples in the low-gigahertz regime, as shown in Figure 35c,d.

Another recent work proposed utilizing a high-directivity microstrip coupled-line directional coupler (with the through port terminated to a match load) for dielectric constant measurements in the low gigahertz regime. The MUT was placed on the coupled-line section of the coupler, and either the coupler’s coupling ($|S_{31}|$) or its isolation level ($|S_{41}|$) was considered as the sensor’s response. The idea behind this work is that putting different MUTs on the coupled line section leads to a change in the coupling coefficient and the isolation level of the coupler. It is noticeable that while, in the works of Piekarz et al. [40,109,110], VNA measurements were required for the odd-mode impedance extraction, and [111] only needs the amplitude of one transmission coefficient; hence, there is no need for a VNA, but instead, either a scalar network analyzer or a frequency synthesizer and a power detector, which make the measurements simpler and lower cost.

**Figure 36:**
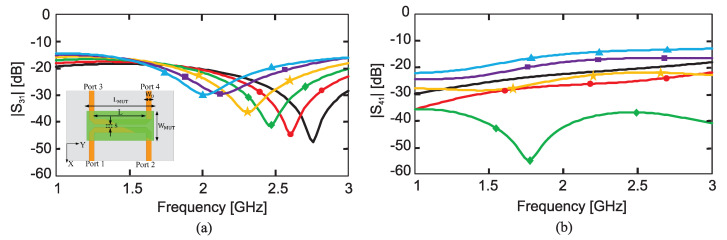
Simulated $|S_{31}|$ (**a**) and $|S_{41}|$ (**b**) of a classical microstrip coupled-line coupler when loaded with different MUTs. Reprinted with permission from Ref. [111].

**Figure 37:**
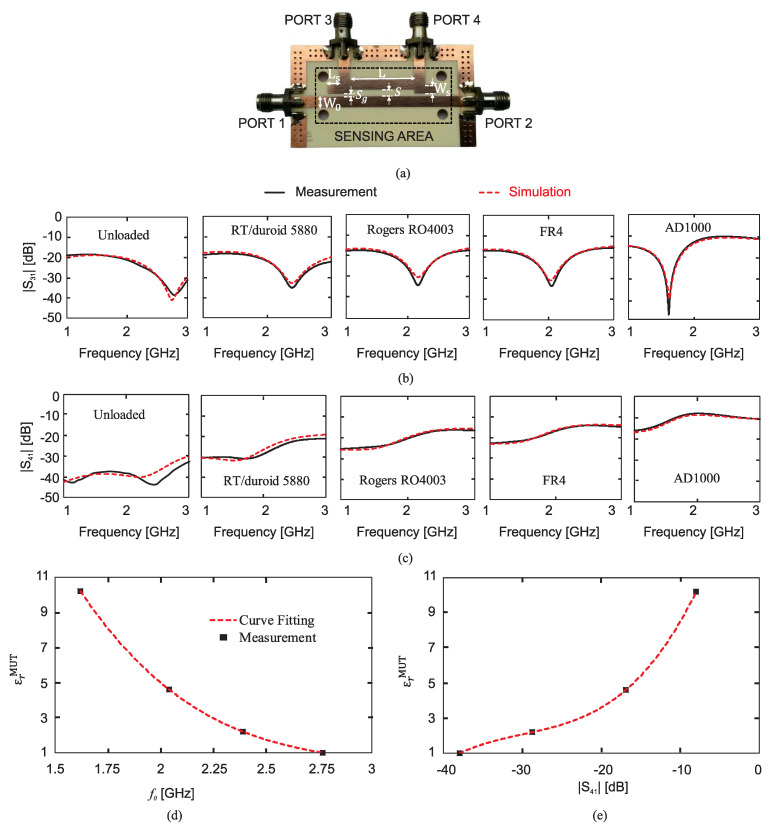
Simulation and measurement result comparison of the fabricated sensor. (**a**) Fabricated high-directivity microstrip coupled-line coupler. Simulated and measured (**b**) $|S_{31}|$ and (**c**) $|S_{41}|$. Dielectric constant of the MUT as a function of the (**d**) zero-coupling frequency ($f_{0}$) and (**e**) $|S_{41}|$ at 2GHz. Reprinted with permission from Ref. [111].

Figure 36a shows the simulated $|S_{31}|$ for a simple microstrip directional coupler loaded with different MUTs. In this figure, the zero-coupling frequency of the coupler ($f_{0}$ at which $|S_{31}|$ is minimum) is 2.76GHz in the unloaded state (where $\varepsilon_{r}^{\mathrm{MUT}}=1$). According to the transmission line theory, zero coupling occurs at the frequency where the effective electrical length of the coupled line section equals an integer number of π. By increasing $\varepsilon_{r}^{\mathrm{MUT}}$ from 1 to 10, $\varepsilon_{r}^{\mathrm{eff}}$ and consequently the effective electric length of the coupled line section increased, and as a result, $f_{0}$ decreased from 2.76GHz to 2GHz, as can be seen in Figure 36a. From this figure, it was concluded that $\varepsilon_{r}^{\mathrm{MUT}}$ can be uniquely retrieved using $f_{0}$. Figure 36b shows the isolation level ($|S_{41}|$) of the coupler for different values of $\varepsilon_{r}^{\mathrm{MUT}}$. However, ref. [111] aimed to use $|S_{41}|$ to detect $\varepsilon_{r}^{\mathrm{MUT}}$ by increasing $\varepsilon_{r}^{\mathrm{MUT}}$, and the trend of $|S_{41}|$ did not monotonically increase or decrease, making the unique extraction of $\varepsilon_{r}^{\mathrm{MUT}}$ impossible. To resolve this problem, by adding two stubs at the ends of the coupled-line section, as shown in Figure 37a, the design of the coupler was modified so that $S_{41}\approx 0$ occurs for $\varepsilon_{r}^{\mathrm{MUT}}=1$. The modified coupler was fabricated and measured with different MUTs. The measured (and simulated) $|S_{31}|$ and $|S_{41}|$ are shown in Figure 37b,c. In Figure 37c, $|S_{41}|$ increases monotonically by increasing $\varepsilon_{r}^{\mathrm{MUT}}$, making the unique retrieval of $\varepsilon_{r}^{\mathrm{MUT}}$ possible. The measurement results of $f_{0}$ and $|S_{41}|$ at 2GHz, for different MUTs, are plotted in Figure 37d,e. It is seen that by increasing $\varepsilon_{r}^{\mathrm{MUT}}$ from 1 (unloaded) to 10.2 (AD1000), $f_{0}$ and $|S_{41}|$ decrease and increase by approximately 42% and 30dB, respectively. Therefore, each of these two parameters can be used to retrieve $\varepsilon_{r}^{\mathrm{MUT}}$ with a high sensitivity. The sensitivity when zero coupling frequency and isolation were used was compared to earlier works based on microstrip resonators and other amplitude-only transmission methods showing a superior sensitivity of the proposed methods. It should be noted that, before using this sensor to retrieve the dielectric constant of unknown MUTs, it needs to be calibrated with some known samples.

### 2.8. Multiplied Sensitivity Aided with Intermodulation Products

An arbitrary sensitivity enhancement is explained in this section with the help of an active circuitry and the nonlinear characteristics of amplifiers. The main idea is based on the mixing product between two resonators. The concept is applicable to a wide range of currently developed resonators when being used as a core of an oscillator. Conventional resonators benefit from variations in the resonance shifts of only one resonance frequency. Conversely, the authors in [41] proposed a cutting-edge scheme wherein the mixing by-products between two resonators were used to create arbitrarily high sensitivities. Assuming that two original sensors produce sinusoidal signals $I_{1}=A_{1}cos\left(\omega_{1}t\right)$ and $I_{2}=A_{2}cos\left(\omega_{2}t\right)$ with frequencies $f_{1,2}=\omega_{1,2}/\left(2\pi \right),\;f_{2}>f_{1}$. Once these signals are summed up and fed into a nonlinear function, e.g., exponential, as follows:

(38) $$I=I_{0}e^{k(I_{1}+I_{2})}=I_{0}e^{k[A_{1}cos\left(\omega_{1}t\right)+A_{2}cos\left(\omega_{2}t\right)]}$$

The resultant components are derived using the Taylor series expansion as follows:

(39) I0eK(I1+I2)=I0∑n=0∞Knn!(i1+i2)n=I0∑n=0∞Knn!∑k=0nnk[A1cos(ω1t)]k[A2cos(ω2t)]n−k

which implies that higher-order components of $\pm m\omega_{1}\pm n\omega_{2}$, m,n∈Z, are generated. Among these, nearby components known as intermodulation products (IMPs) are located at:

(40) $$\begin{array}{cc} 3^{rd}\;IMP\;\;\;\to \;\;\;\;\;\;\;\;\;\;\;\;\;\;\;2f_{1}-f_{2} & =f_{1}-\Delta f_{0}\\ 5^{th}\;IMP\;\;\;\to \;\;\;\;\;\;\;\;\;\;\;\;\;3f_{1}-2f_{2} & =f_{1}-2\Delta f_{0}\\ 7^{th}\;IMP\;\;\;\to \;\;\;\;\;\;\;\;\;\;\;\;\;4f_{1}-3f_{2} & =f_{1}-3\Delta f_{0} \end{array}$$

**Figure 38:**
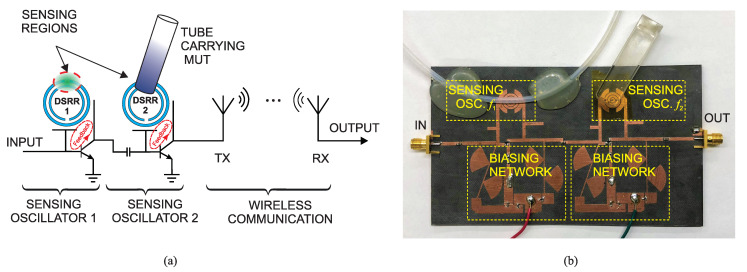
The schematic of t0he intermodulation-based microwave sensor. (**a**) Schematic of the intermodulation-based sensor and wireless communication. (**b**) Fabricated sensor. Reprinted with permission from Ref. [41].

       ⋮             ⋮

(41) $${\left(2k+1\right)}^{th}\;IMP\;\;\;\to \;\;\;\left(k+1\right)f_{1}-kf_{2}=f_{1}-k\Delta f_{0}$$

where the initial frequency separation is defined as $\Delta f_{0}=f_{2}-f_{1}$. This clearly suggests that variations in the original frequencies $\left(f_{1},f_{2}\right)$ can be multiplied by integer values when IMPs are monitored. A supremely elaborated description is given in [41], where two similar microwave planar resonators operating at $f_{2}=5\;\mathrm{GHz}$ and $f_{1}=4.75\;\mathrm{GHz}$ are used as the core sensing elements. The resonators are comprised of two concentric SRRs, also known as double split-ring resonators (DSRRs), which are highly coupled to create an ultra-high-sensitivity region. In order to generate a signal with the frequencies $f_{1}$ and $f_{2}$, the resonators are employed as the tank of oscillators. Each resonator is utilized in the feedback path of a regenerative amplifier, and its operation is transformed into a feedback oscillator once the resonator loss is fully compensated. In order to couple these two signals in a nonlinear component, the authors in [41] devised a mixing technique to couple the two individual signals as shown in Figure 38a.

In this design, the second sensing oscillator is used as both generator of $f_{2}$ and the mixing element for the signal $f_{1}$ being injected from the first stage. The second stage needs to operate at a nonlinear region for the bandwidth of interest to allow for mixing products being produced with reasonable gain at the output. This mixer design is followed by a pair of wide-band high-gain linear antennas for transmission purposes. The use of an antenna illustrates that the proposed sensing technique can be integrated with peripheral electronic receivers to enable radio frequency identification (RFID).

The experimental verification of the mixer sensor is conducted using a PTFE tubing $\left(OD=1/8^{\prime \prime }\right)$ as a carrier, and is configured on the first stage with a lower oscillation frequency $f_{1}$ to freely shift without interfering with $f_{2}$. The mixer sensor is fabricated on Rogers RO5880 substrate (see Figure 38b) and is connected to a pair of wide-band slot bow-tie antennas. Once the antennas are separated by 30 cm, the received power at the receiving antenna is measured using a power meter. The sensor is examined with water as the external analyte to exhibit its fundamental functionality. Since the tube is directly affecting the first stage, the oscillation frequency $f_{1}$ is downshifted. However, the second stage with $f_{2}$ is located at a distance with respect to the first resonator, and then is left unaffected. IMPs with orders of 3, 5 and 7 have frequency differences of $\Delta f_{0}$, $2\Delta f_{0}$ and $3\Delta f_{0}$ from $f_{1}$, as well as reveal correspondingly increased downshifts. This represents that the proposed sensor allows for up to 3-fold sensitivity enhancement at a lower frequency, which simplifies the post-processing in commercial applications.

**Figure 39:**
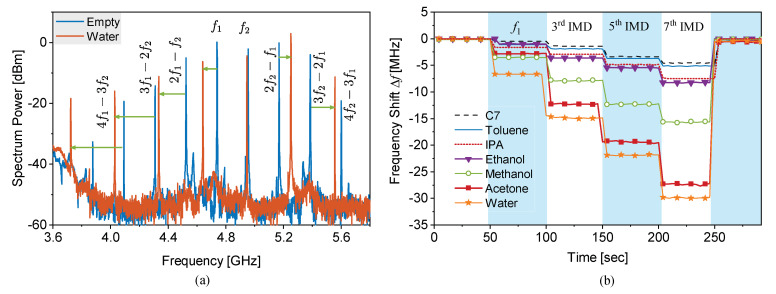
Intermodulation-based sensor response. (**a**) IMPs in the spectrum analyzer for $f_{2}=5\;\mathrm{GHz}$ and $f_{1}=4.75\;\mathrm{GHz}$. (**b**) Material characterization and sensitivity comparison using 3rd, 5th, and 7th IMPs. Reprinted with permission from Ref. [41].

In another experiment, the sensitivity enhancement is compared between $C_{7}$, toluene, IPA, ethanol, methanol, acetone, and water, as shown in Figure 39b. Assuming that $f_{1}$ undergoes a certain change in the frequency, the higher-order IMPs represent multiplied shifts that are consistent for a wide range of permittivity ranging from single-digit $\varepsilon_{r}$ for $C_{7}$ and toluene to a larger value for water.

A reasonable concern for such sensors is the possible accumulation of $f_{1}$, $f_{2}$ phase noise and its appearance in the IMP with the same enhancement factor. In this regard, the authors in [41] have well studied this issue and reported a possible and feasible case considering which such a disturbing factor is avoided in the sensor response. It has been shown that the phase lag between two incoming signals (currents) need to be infinitesimal, which is realized by shortening the distance between the output of the first stage and the input of the second stage.

## 3. Resolution Enhancement

Microwave sensors can be in either transmission type or reflection type depending on the application and the number of ports required. Each resonator can be represented with a series or parallel resonator and shown with lumped circuit components [112]. In this regard, the loss of MUT plays an important role in the sharpness of the sensor profile $(S_{21}$ or $S_{11})$. It is evident that sharper profiles are easier to distinguish and therefore favorable to be used in high-end applications. However, material loss degrades the sensor performance with lowering the quality factor of the transmission-based sensors and matching of reflection-based ones. In this regard, the sensor resolution denotes how separate sensor responses become as a result of a given change in the MUT. In other words, once the $S_{21}$ profile of a sensor shifts due to a change in the environment, high-resolution sensor results in low-to-no overlap between the original and shifted $S_{21}$ profiles. This section discusses various methodologies that can be applied to a given resonator to improve its resolution (profile sharpness).

### 3.1. Active Transmission-Based Sensors

**Figure 40:**
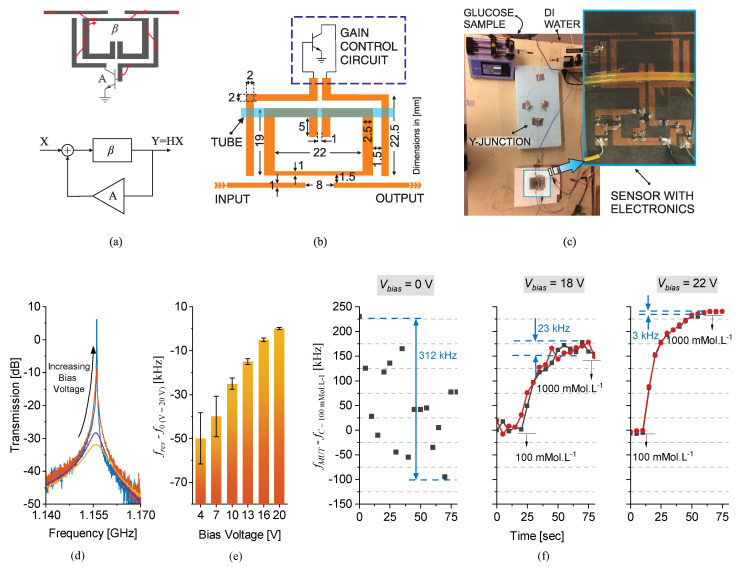
Active sensor design, schematic and preliminary results: (**a**) feedback schematic of an active resonator; (**b**) schematic of the active resonator with dimensions; (**c**) Sensing setup for glucose monitoring with two syringe pumps; (**d**) bias voltage impact on the transmission response; (**e**) bias voltage impact on the sensor response uncertainty; (**f**) monitoring glucose concentrations ($100\to 1000\;\mathrm{mg}\;\cdot \;{\mathrm{dL}}^{-1}$) with different states of the active sensor $(V_{bias}=0,18,22$ V) with two separate measurements (black and red lines). Reprinted with permission from Ref. [46].

A wide range of microwave sensors are employed in two-port configuration, which makes them suitable for being used as part of an amplifier or oscillator. Therefore, this section only discusses the high-resolution sensors with transmission response. The sensor loss rises from different sources including MUT loss, the proximity of external objects, dielectric/conductor loss, and electromagnetic propagation. Regardless of the loss source, the transmission profile undergoes a degradation in the sharpness. Having said that, our focus in this part is on material loss. One approach to still elicit information from a lossy MUT is to compensate the loss of the sensor. This has been meticulously elaborated in [46] followed by several other similar works in this regard [42,43,113,114]. The authors proposed an active sensor as a viable solution to cope with the losses in the system. In an active sensor, electronics are involved to compensate the resonator loss. Figure 40a represents a simplified version of the proposed system where the resonator is coupled to an amplifier in the feedback path to generate a regenerative amplifier. The transfer function of a feedback system with amplifier gain *A* and resonator transfer function β is as follows:

(42) $$H=\frac{Y}{X}=\frac{\beta }{1+A\beta }$$

which not only relies on the forward path β, but is also modified by the amplification coefficient (1+Aβ). The optimum conditions for the sensor response include:

- Gain *A* to compensate the resonator loss β; i.e., $\left|A\right|\approx \left|\beta \right|$
- The wrapped phase of resonator β to equal the amplifier phase; i.e., ∡A=∡β

As such, the resonator loss is controllable and the system loss issue, mainly derived by MUT loss, can be arbitrarily fixed. In this regard, partial compensation is enough, beyond which the sensor enters oscillation and the operation mode changes. An edge-folded SRR was designed in [46] on a Rogers RO5880 substrate with the dimensions given in Figure 40b. The resonator at the forward path of a regenerative amplifier is loss-compensated with a bipolar transistor (i.e., NE68033) and the phase modification is implemented with the help of transmission lines. The sensor resonates at 1GHz with $Q_{Passive}=190$. In order to verify the active sensor, a PTFE tube ($ID=1/16^{\prime \prime }$) is used that covers the resonator gap as well as the SRR to improve the sensitivity. The active circuit is triggered with a bias voltage that essentially controls the transistor’s operation. Increasing the bias voltage allows for higher transistor current that results in an increased $S_{21}$ peak as shown in Figure 40d. It is evident that the sensor resolution is substantially improved with more than 20-fold Q-factor enhancement (i.e., $Q_{Active}\approx 3850$). The high Q-factor ensures that variations in the measured resonance frequency because of a change in the effective dielectric constant, here due to glucose, that can be discerned with smaller steps. The incertitude in the measured resonance frequency is demonstrated by an error bar in Figure 40e at different bias voltages. It is noteworthy that the central frequency relocates slightly along with the bias voltage because the active circuit load on the resonator depends on the operational mode of the transistor. Moreover, the error bars depict the fluctuations in the frequency of resonance decreasing as the loss-compensation improves at higher bias voltages. As a result, a more precise sensor performance with low uncertainty with active sensor design. A more practical study is conducted on the sensor robustness at different loss-compensation states with sensing glucose concentrations in an aqueous solution. The proposed active sensor is used as a glucose-sensing device for noninvasive monitoring. To this aim, an ultra-high sensitivity is required to distinguish ≤ 200 ppm glucose concentration change (i.e., $1\;\mathrm{mMol}\cdot L^{-1}\approx \;18\;\mathrm{mg}\cdot {\mathrm{dL}}^{-1}$) in an aqueous solution. Two syringe pumps with variable flow rates are utilized to combine DI water with a highly concentrated glucose solution (i.e., $1000\;\mathrm{mMol}\cdot L^{-1}$) to achieve intermediate concentrations, as shown in Figure 40c. Glucose concentration is varied from $100\;\mathrm{mMol}\cdot L^{-1}$ to $1000\;\mathrm{mMol}\cdot L^{-1}$ and monitored with the sensor resonance frequency continuously within 75 s. Passive mode of the resonator, shown with $V_{bias}=0\;V$, yields highly scattered measured resonance frequencies due to a large 3 dB bandwidth, which allows for frequency span to undergo white noise as shown in Figure 40d. At higher bias voltages (e.g., $V_{bias}=22\;V$), in contrast, the sensor loss compensation diminishes the white noise impact on the transmission profile, and subsequently, on the resonance frequency detection. It is clearly shown that the uncertainty in the frequency detection reduces from 312kHz down to 3kHz. This low frequency fluctuation reflects the high resolution of the sensor that helps discriminate smaller glucose concentration variations. Subsequently in this work [46], it is shown how this very sensor can be used to distinguish much smaller glucose levels in the range suitable for the human body (3–6.5 $\mathrm{mMol}\cdot L^{-1}\approx$ 50–130 $\mathrm{mg}\cdot {\mathrm{dL}}^{-1}$).

### 3.2. Active Reflection-Based Sensors

**Figure 41:**
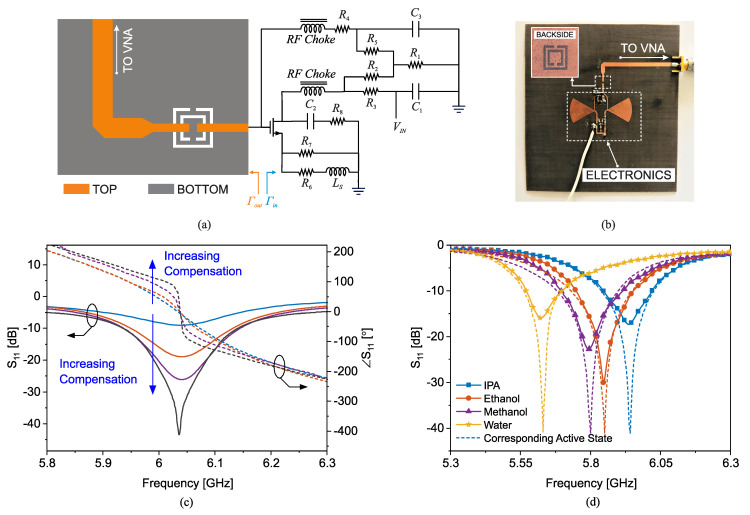
Reflection sensor loss-compensated design with preliminary results: (**a**) schematic of the active reflection-based sensor; (**b**) fabricated sensor prototype; (**c**) Measured amplitude and phase of reflection parameter $S_{11}$ with different loss compensation states from blue (no compensation) to black (high compensation); and (**d**) measured $S_{11}$ for different Materials including IPA, ethanol, methanol and water. Reprinted with permission from Ref. [115].

In addition to transmission-based sensors, single-port resonators were also loss-compensated to be used as reflection sensors based on reflection scattering parameter ($S_{11}$) as introduced in [115]. This type of sensor is suitable for applications that require a single port and hence low computational effort in post processing. A conventional CSRR is etched from the ground plane as a highly sensitive region for dielectric material characterization, and is excited by a transmission line on the other side of the substrate as shown in Figure 41a. The sensor $S_{11}$ is designed to have low reflection at the frequency of ∼6GHz; a fairly high frequency is chosen for a decent sensitivity. This resonance becomes impacted by the loss of MUT and the depth of $S_{11}$ degrades in sharpness. Hence, the resonator loss is retrieved by an active circuit. In this case, because of the single-port characteristic of the resonator, a reflection-based negative resistance is generated with the help of ATF34143 FET. The DC biasing is implemented by a feedback from drain to gate through a voltage division ($R_{1}/R_{2}$) and the resistive source ($R_{6}=R_{7}$) results in a negative $V_{gs}$. As such, the biasing is possible with a single bias voltage $V_{IN}$. The negative resistance of the circuit depends on the Barkhausen criterion, where oscillations are triggered at the gate node when the reflection parameter seen from the input of the circuit ($\Gamma_{in}$) and the output of the CSRR ($\Gamma_{out}$) meets the following condition:

(43) $$\left|\Gamma_{in}\right|\times \left|\Gamma_{out}\right|>1$$

However, partially compensating the sensor loss is used to use utilize the sensor in the resonance mode without entering the oscillation regime. A fabricated sensor is shown in Figure 41b with the CSRR on the ground side represented as the inset. Retrieving the resonator loss translates to recovering the stored power in the resonator by changing the input impedance of the active circuit to complex-conjugately match with the resonator. Therefore, the reflections from the sensor minimizes, which is elaborated with the increased depth of $S_{11}$ in Figure 41c. With higher bias voltages, the amplitude of reflection decreases and the corresponding phase response becomes sharper at the frequency of resonance. A performance examining test is conducted with the sensor being loaded by various common liquids including IPA, ethanol, methanol, and water flown into a PTFE tubing. A typical sensor reflection response is given in Figure 41d with solid lines. Once the active circuit is triggered, the loss-compensated profiles with much higher resolution and sharpness are generated as shown with dashed lines. This implementation confirms that the sensor response with a single port of excitation can be configured in a negative feedback structure to restore the lost power. This structure can then be used for sensing much smaller variations in an MUT.

### 3.3. Oscillator-Based Sensors

**Figure 42:**
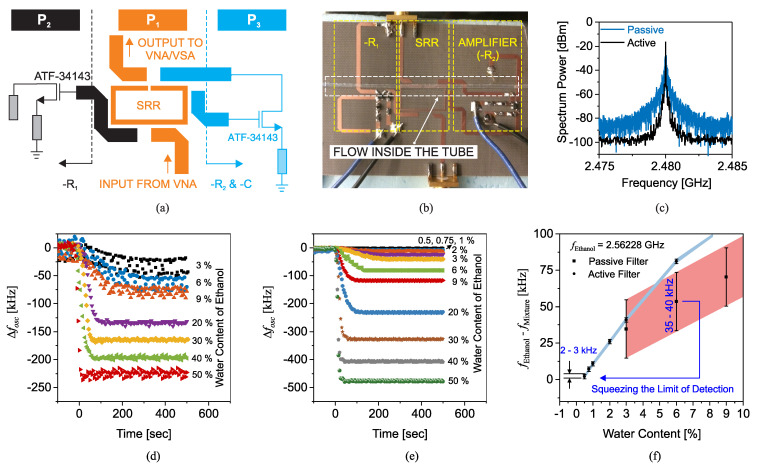
Active sensor with active tank: design, schematic and sensing results: (**a**) schematic of the oscillator with active tank; (**b**) fabricated oscillator with active tank; (**c**) spectrum power with oscillator tank in passive/active modes; (**d**) measured oscillation frequencies with concentrated water in ethanol with passive tank; (**e**) measured oscillation frequencies with concentrated water in ethanol using active tank; and (**f**) comparing the oscillation frequency stability in both a passive and active tank for a given MUT. Reprinted with permission from Ref. [43].

Another important type of loss-compensation is full compensation, which allows for not only the sensor response retrieval but also the sensor to be used for single-port applications without excitation. Once the lost power of a resonator at the frequency of resonance ($f_{res}$) is fully retrieved, the resonance behavior turns into oscillation. With this method, one can benefit from the signal generated at $f_{res}$, which is still tightly linked with the resonator parameters (e.g., resistance, capacitance and inductance). The prototype of a planar oscillator sensor is debuted in [43], wherein an SRR at $f_{res}=2.4\;\mathrm{GHz}$ is connected to a negative resistance unit $R_{1}$, as shown in Figure 42a. A source degenerated ATF-34143 metal–oxide–semiconductor field effect transistor (MOSFET) provides the negative resistance to remove the SRR loss. This method enables a high-resolution sensing paradigm since the whole-transmission/reflection from a resonator is reduced to a single frequency.

In the study of an oscillator, the operational frequency is prone to fluctuations in the transistor current that can be originated from white noise, flicker noise, etc. When there is no apparent variation in the MUT, these fluctuations determine the sensor’s bit resolution (i.e., limit of detection, lower than which the sensor data are noisy). Quantifying the magnitude of such variations depends on the impedance analysis of a parallel combination between the resonator circuit model that is supposed to be a parallel RLC (R: resistor; L: inductor; C: capacitor), the load impedance $R_{Load}$, and the negative resistance $-R_{N}$. The admittance seen from the input of the sensor is given below:

(44) $$Y_{in}=\frac{1}{R+R_{Load}}\left(1\pm 2jQ\frac{\Delta f}{f_{0}}\right)-\frac{1}{R_{N}}$$

where *Q* is the quality factor and Δf denotes the frequency offset from the center frequency $f_{0}$. In oscillation, impedance matching leads to impedance removal, which reduces (44) to:

(45) $$Y_{in}=\frac{\pm 2jQ}{R+R_{Load}}\;\cdot \;\frac{\Delta f}{f_{0}}$$

The uncertainty in the frequency of oscillation is elaborated by the phase noise (PN) of a system, which is computed after manipulating (45) in 1 Hz bandwidth as follows:

(46) $$L\left(f\right)=\frac{kT}{2Q^{2}P{\left(\frac{f_{0}}{f}\right)}^{2}}$$

where $k=1.38\times 10^{-23}\;J/K$ is the Boltzmann’s constant, T[K] is the temperature and P[W] is the output power. Equation (46) suggests that the sensor PN can be improved with higher output power *P* and improved quality factor *Q*. As a result, one of the main tools to increase the sensor stability is to employ high-*Q* resonators. In this regard, the authors in [43] have elaborated in detail that the resonator loss can be compensated to be used in the tank of an oscillator. This loss-compensation leads to an active SRR with improved quality factor that is implemented with a regenerative amplifier design in blue (see Figure 42a). This compensation can also be modelled with a negative resistance as described in Section 3.1.

A fabricated sensor with both a negative resistance circuit ($-R_{1}$) as well as the regenerative amplifier ($-R_{2}$), both coupled on the main SRR, is shown in Figure 42b. The SRR is chosen to be narrow in width to be highly impacted by the microfluidic tube above it. The measurement of the single-port oscillator sensor with a vector spectrum analyzer is depicted in Figure 42c, in both cases, where the oscillator uses the tank in the passive mode, as well as the sensor with the active tank. It is clear that the spectrum of the sensor becomes narrower, which implies that less noise is aggregated on the surrounding offset frequencies of the oscillator. In addition, the output power at the oscillation frequency improves from −27.5dB to −16.17dB due to the active tank. Furthermore, the spectrum is less noisy when identical offset frequencies are compared. This improvement in the spectrum translates to the sensor’s stability in the generated frequency of operation, which then dictates the fluctuations in the sensor operation. A higher stability in the frequency response results in the higher resolution of the sensor.

The verification of the proposed design with liquid characterization is performed next. In this experiment, water in ethanol solutions are injected inside the microfluidic tube on the SRR, whose concentrations are determined by two syringe pumps controlling DI water and ethanol, respectively. Injections start with ethanol (as a starting point on the curve) and followed by water to obtain the desired concentrations ranging from 50%→3%. It is evident that the passive tank with a relatively low quality factor results in scrambled frequencies. The oscillation frequency difference between ethanol and the concentrated solution ($\Delta f_{osc}$) is shown in Figure 42d. In order to evaluate the bit resolution of the sensor, concentration increments smaller than 3% are difficult to discriminate. However, the same experiment with the active tank is represented in Figure 42e, where measured oscillation frequencies are highly stable. This robustness allows characterizing even smaller quantities of water in ethanol down to the 0.25% level, which clearly demonstrates that the oscillator with an active tank results in high-resolution sensing. A summary of this measurement is demonstrated in Figure 42f, which showcases the oscillation frequency shift with respect to the water content for both oscillator modes. The sensor response with the tank in the passive mode is associated with a high incertitude of ±20kHz. This uncertainty is significantly improved towards a much smaller frequency deviation ±1.5kHz with the tank being in active mode. This very feature also reduces the lowest detectable concentration of water in ethanol in a given sensing configuration by a factor of ∼12 ( from 3%→0.25%). The proposed active tank structure can be implemented to different resonator designs, including but not limited to, planar and substrate integrated waveguide-based sensors.

### 3.4. Differential Measurements with Cross-Mode Transmission Coefficient

**Figure 43:**
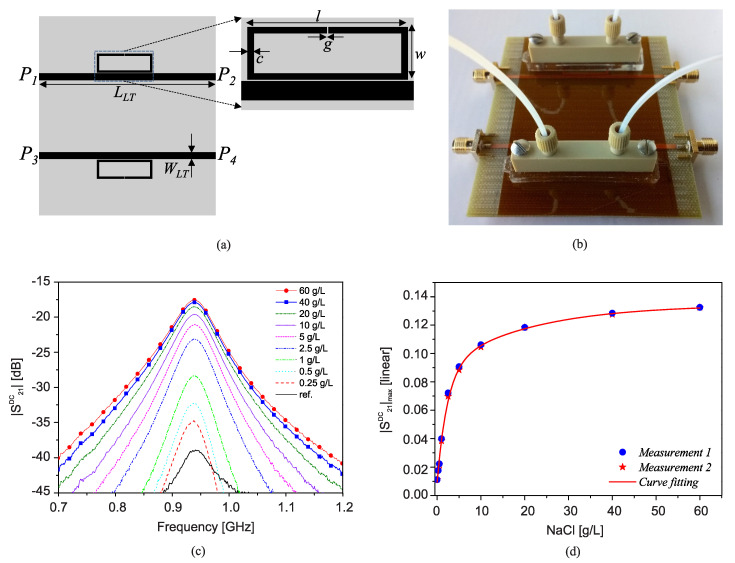
Topology of the differential sensor with the effects of NaCl concentration on the cross-mode transmission coefficient: (**a**) topology of the microwave structure of the SRR-based differential sensor; (**b**) photograph including the microwave structure, the fluidic channels (plus mechanical accessories) and connectors. Dimensions (in [mm]) are: $L_{LT}$ = 76.5, $W_{LT}$ = 2.79, *l* = 24, *c* = 1, *w* = 8.17, *g* = 0.2. The ground plane is depicted in grey. The sensor was implemented in the FR4 substrate with the dielectric constant $\varepsilon_{r}$ = 4.4 and thickness *h* = 1.6 mm; (**c**) dependence of the cross-mode transmission coefficient on frequency; (**d**) relation between sodium concentration and ${\left|S_{21}^{DC}\right|}_{max}$ in linear form. In (**d**), two measurement campaigns have been carried out in order to ensure repeatability. Reprinted with permission from Ref. [117].

An approach that provides very good resolution such as differential sensing functions by means of resonator-loaded lines, as shown in Figure 43a,b. In such sensors, a pair of transmission lines is loaded with the sensitive elements, planar resonators. The lines and resonators are identical, and therefore, any differential output variable should be null provided symmetry is not perturbed. However, by loading one of the resonators with the so-called reference (REF) material, and the other one with the material under test (MUT), symmetry is disrupted (as far as the REF and MUT are different), and a differential output variable should be generated. It has been found that the cross-mode transmission coefficient is a very useful output variable in this kind of sensors [52,116,117,118,119]. Indeed, if the two resonator-loaded sensing lines are uncoupled, the cross-mode transmission coefficient is given by [120]

(47) $$S_{21}^{DC}=\frac{1}{2}\left(S_{21,\mathrm{MUT}}-S_{21,\mathrm{REF}}\right)$$

and it is therefore a canonical differential variable, as far as it is proportional to the difference between the transmission coefficients of the individual lines. Ideally, under perfect balance, the cross-mode transmission coefficient should be null, however, in practice, perfect balance is impossible to achieve, and the transmission coefficient is finite. Indeed, an indication of the level of quality of this type of sensors is given by the magnitude of the cross-mode transmission coefficient under perfect balance. To illustrate the potential of the approach, we report an example of a differential-mode sensor devoted to the measurement of the concentration of electrolytes in aqueous solutions. The sensor, depicted in Figure 43a, is equipped with fluidic channels for liquid injection (the details can be found in [117]). Such channels are placed on top of SRRs, the sensing resonators. The sensor acts as a comparator able to detect differences between the REF liquid (pure DI water) and the MUT (or liquid under test—LUT), a solution of electrolytes in DI water. The resolution of the sensor is given by the minimum concentration of electrolytes that can be resolved.

Figure 43c depicts the cross-mode transmission coefficient for different values of the concentration of electrolytes (particularly NaCl), as well as the value of the maximum magnitude as a function of the concentration of electrolytes. The sensitivity (see Figure 43d) is very high in the limit of small perturbations, and it progressively decreases (the maximum value is found to be $0.033\;{\left(g/L\right)}^{-1}$). However, it is relevant that the sensor is able to detect concentrations of NaCl as small as 0.25 g/L (note that the corresponding curve in Figure 43c perfectly differentiates from the one corresponding to the sensor loaded with DI water in both channels). Thus, sensor resolution is 0.25 g/L, which can be considered to be a very good value.

## 4. Robustness Enhancement

Conventional microwave sensors are suffering from a low-to-moderate quality factor. This issue reduces their range of application when they are employed in cutting-edge sensing mode. In order to overcome the issue of the low-quality factor, active resonators, oscillators and mixers are proposed and decently elaborated as in [41,42,43,46,113,114] to compensate the sensor loss and improve the response. This very feature of loss-compensation increases the sensor’s sensitivity to the surrounding medium so that permittivity variations in further distances can also be detectable. As a result of MUT detection and characterization at broader physical distances, the sensor becomes susceptible to the sensing environment, which concedes impacts from undesired materials or minor temperature/humidity variation. This is the main motive to improve the robustness of microwave sensors empowered by an active circuitry to still benefit from their high resolution. In this section, different novel approaches are introduced to help minimize unwanted impacts on sensitive sensors.

### 4.1. Robustness to Distance Using Fuzzy Neural Network

**Figure 44:**
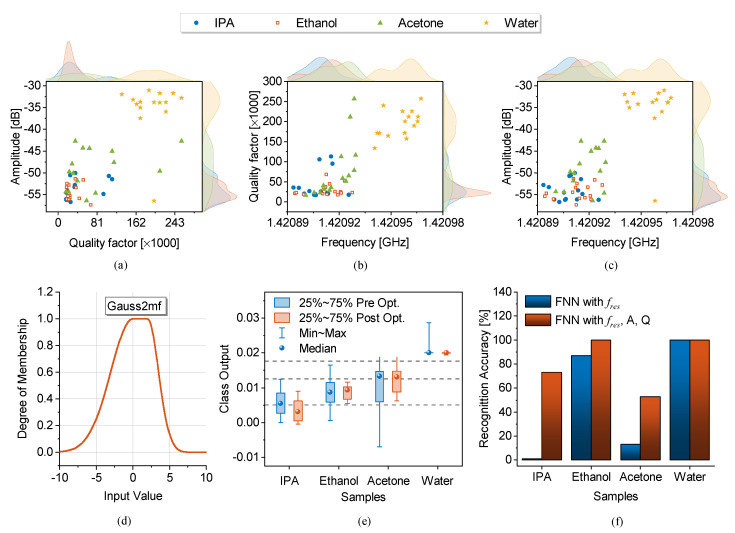
Sensor response for various MUT location and classification results. Sensor response comparison exposed to IPA, ethanol, acetone, and water between (**a**) Amplitude and Q-factor, (**b**) Q-factor and frequency, (**c**) Amplitude and frequency. (**d**) Gaussian membership function in Fuzzy neural network example, (**e**) Sample classification results without (blue) and with (red) optimization, (**f**) Comparing common MUT recognition accuracy when only $f_{res}$ is used as apposed to incorporating all $f_{res},A,\;\mathrm{and}\;Q$. Reprinted with permission from Ref. [32].

One of the main characteristics associated with planar sensors is their sensitivity to effective permittivity variation, which is affected by the distance of MUT with respect to the sensor surface. It is clear that the sensing element of microwave planar sensor pursues MUT once it is in the range of fringing fields emanated from the resonator. It has been shown in active sensors that the fringing fields are extendable in range with the help of loss compensation [46,113,114]. This range extension is employed to help sensing MUTs located at a distance away from the sensor surface [32]. A meandered type half-wavelength resonator is used to achieve a compact sensor size operating at ∼1.3GHz. Various chemicals including 2-Isopropanol (IPA), ethanol, acetone and water were introduced to the sensor inside a plastic cylindrical container. To examine the sensor response with respect to the physical distance between the MUT and the sensor (Δd), the container dislocates manually around a central point that is one wavelength (Δd=λ≈25mm) away from the sensor. It is noteworthy that such a high distance in sensing is only feasible because of the loss compensation in the resonator. The transmission response of the sensor is summarized with three orthogonal (principal) components of amplitude, quality factor, and resonance frequency. The comparison between these parameters measured by 15 times for all liquids is given in Figure 44a–c. Scrambled symbols denote the spread of the corresponding quantity for a given MUT as a result of slight dislocation. The projection of the obtained transmission characteristics on amplitude vs. Q-factor, Q-factor vs. frequency and amplitude vs. frequency vectors show an intermingled relationship any dual sensor output. Therefore, MUT characterization becomes highly challenging. The authors in [32] debuted and developed the application of machine learning algorithms in sensing towards improving the sensor performance. As of the algorithm, the fuzzy neural network (FNN) is used as a reliable means of mapping several inputs to a target output(s). The input parameters of an FNN are linked through arithmetic particulates of AND and OR based on general rules with the following format:

$$x=A_{i}\Rightarrow y=f_{i}\left(x,a_{i}\right)=a_{0}+a_{i}^{T}x\left(*\right),\;\;\;\;\;i=1,2,\ldots ,c.$$

where $A_{i}$ is the fuzzy set designed in the multivariate input space and $f_{i}$ in the local linear model. Logic models denoted by (*) are able to nonlinearly map between the input and output. Furthermore, the parameters of condition (i.e., fuzzy set $A_{i}$) and conclusion (i.e., $a_{0},a_{i}$) can be optimized (learned).

The FNN input consists of 3 values per sample measured 15 times, totaling 60 inputs considering 4 MUTs that are supposed to be clustered into 4 categories. In this analysis, a linear model is used as follows:

(48) $$\hat{y}=\sum A_{i}\left(x\right)\;\cdot \;\left(a_{0}+a_{i}^{T}x\right)$$

where $A_{i}$ as the membership function (Gauss2mf) is composed of two Gaussian distributions, typically constructed by clustering techniques [121,122], with the following generic forms:

(49) $$f_{1i}\left(x\right)=\left\{\begin{array}{c} e^{\frac{-{\left(x-c_{1i}\right)}^{2}}{2\sigma_{1i}^{2}}}\;\;\;\;\;\;\;\;\;\mathrm{if}\;\;x⩽c_{1i}\\ 1\;0\;\;\;\;\;\;\;\;\;\;\;\;\;\;\;\;\mathrm{otherwise} \end{array}\right.$$

(50) $$f_{2i}\left(x\right)=\left\{\begin{array}{c} e^{\frac{-{\left(x-c_{2i}\right)}^{2}}{2\sigma_{2i}^{2}}}\;\;\;\;\;\;\;\;\;\mathrm{if}\;\;x⩽c_{2i}\\ 1\;\;\;\;\;\;\;\;\;\;\;\;\;\;\;\;\;\mathrm{otherwise} \end{array}\right.$$

where $\sigma_{1i},\sigma_{2i}$ and $c_{1i},c_{2i}$ are the spread and prototype of the $i^{th}$ fuzzy set, respectively. Then, Gauss2mf is given by $A_{i}\left(x\right)=f_{1i}\left(x\right)f_{2i}\left(x\right)$, which is graphically shown in Figure 44d as an example. Essentially all measured values for the three main contributing factors of $A,f_{res},$ and *Q* are separated into different classes with respect to the corresponding membership function. The parameters of the linear model in a collection of input–output as $(X_{in},T_{k}),\;k=1,2,\ldots ,N$ are estimated by minimizing the mean squared error (MSE) as follows:

(51) $$MSE=\frac{1}{N}\sum_{k=1}^{N}{\left({\hat{y}}_{k}-T_{k}\right)}^{2}$$

Training, validation and test sets take 70%, 15%, and 15% of the input set, respectively. Reliable training is conducted through averaging 15 individual training through single-point out method because of the limited number of data points. The output target is chosen to be arbitrarily chosen discrete values of 0,0.01,0.015,0.02 as shown in Figure 44e. The blue box plots represents predicted outputs, which are supposed to be within dashed lines as boundaries of the adjacent classes. Whiskers demonstrate the minimum and maximum of the mapped output. This classification is further improved by optimizing the parameters based on combined methods of back-propagation gradient-decent and least square error that is shown with red box plot. It is evident that the data points residing outside of the corresponding boundary are pulled inside after optimization, thereby improving the classification accuracy. A comparative analysis is given in Figure 44f, which showcases the result of FNN with a single parameter input, here frequency $f_{res}$, compared with an FNN using all three parameters of $\left(f_{res},A,Q\right)$. It is clear that the prediction accuracy, which is the division of correct classification over total number of inputs, is significantly improved for IPA and Acetone when using all three orthogonal parameters rather than only frequency. This improvement results in an average of 81.5% prediction accuracy for the latter compared with only 49.5% whilst using the former input set. In general, the proposed FNN is used to enable the correct prediction of common liquids with considerably high accuracy (100% for water) even though the sample location is noisy, which depicts the robustness of the machine-learning-assisted sensor in dealing with an unstable environment.

### 4.2. Neutralizing Environmental Impacts with Machine Learning

**Figure 45:**
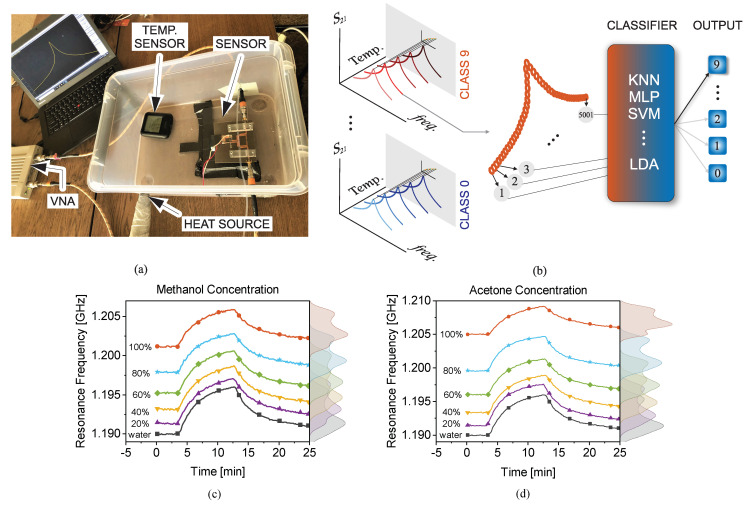
Temperature modulation of the active sensor loaded with MUT. (**a**) sensing setup with sealed box to control the humidity; (**b**) schematic of the proposed architecture using machine learning for classification. Measured sensor response overlap for various concentrations of water in (**c**) methanol and (**d**) acetone undergoing temperature cycle $\left(25\to 55^{\circ }\right)$. Reprinted with permission from Ref. [49].

An example of environmental interference (noise) is the presence of undesired materials within the sensing range. While practical situations involve a complex matrix, a sensor needs to be selective to the desired MUT. Machine learning has been found to be lucrative in enabling selectivity in microwave sensors, as shown in [123], where a convolutional neural network is used to process the sensor response with respect to its pre-trained repository. Another aspect of environmental noise on the microwave sensor response is explained in this section as the temperature effect. Temperature, similarly to other ambient factors including humidity, pressure, proximity, force, etc., changes the effective presence of an MUT in material characterization. This is mainly due to the noninvasive characteristic of planar sensors that allow for materials to be influenced by the ambient [124]. The authors in [49] demonstrated an SRR operating at ∼1.2GHz, which is used for material characterization using a PTFE tubing. The sensor is employed in a sealed box that is connected to a heat source and the box temperature is monitored with a commercial thermometer as shown in Figure 45a.

**Figure 46:**
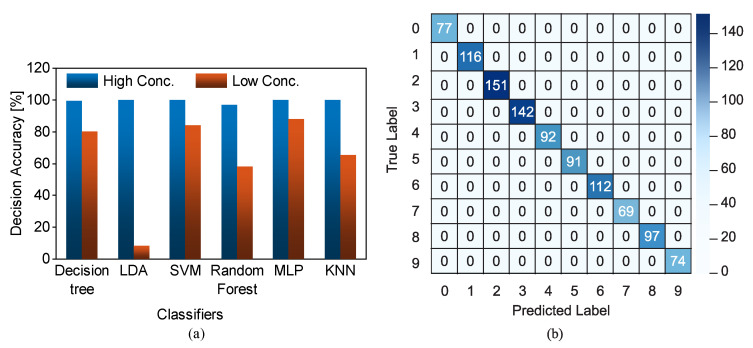
Sensor response classification results with respect to temperature: (**a**) comparison between different classifiers for both high and low concentrations; (**b**) confusion matrix for classification results of methanol in water (0→4) and acetone in water (5→9). Reprinted with permission from Ref. [49].

A temperature cycle is applied to the box, which impacts both the sensor and the MUT. It is well known that the temperature contributes to the effective permittivity of materials as follows [125]:

(52) $$\frac{\left(\varepsilon_{r}-1\right)\left(2\varepsilon_{r}+1\right)}{9\varepsilon_{r}}=\frac{4\pi \rho N_{A}}{3M}\;\cdot \;\left(\alpha +\frac{\mu^{2}g}{3k_{B}T}\right)$$

where *M* is the molecular weight, ρ is the density, α is the molecular polarizability, $N_{A}$ is Avogadro’s number, μ is the dipole moment of the molecule, *k* is the Boltzmann’s constant and *g* is a factor to characterize the orientation of neighboring molecules. Expression (52) elaborates that the permittivity reduces with elevated molecule interaction and lowered density at higher temperatures. This leads to an increase in the measured resonance frequency when the material warms up. The challenge is that when several materials are characterized, their $f_{res}$ variations are intertwined such that inferring the presence of an MUT is not practically possible with respect to only monitoring $f_{res}$. This behavior is the result of the significant impact of the environment on the sensing platform in an uncontrolled medium. The authors in [49] showed that the use of an artificial neural network in managing the situation with proper allocation of sensor responses to each MUT. In this regard, they proposed a method that involves correlating measured sensor data as the input set to a desired MUT as the target class. In each class, varied transmission profiles ($S_{21}$) with 5001 points each are measured. Inspired by [32], the authors in [49] exploited deeper levels of the measured $S_{21}$. In this work, the whole magnitude of transmission response $|S_{21}|$ over 5001 frequency points are considered as shown in Figure 45b. The sensor is exposed to a wide range of permittivity values mixing methanol and water to arrive at a 20% increment in concentrations from 0→100%. Each sample is tested individually and the overall sensor resonance frequency variation over time is shown in Figure 45c. The temperature of the box increases from a room temperature of $25{\;}^{\circ }C$ up to $50{\;}^{\circ }$C, which increases the PTFE tubing, MUT, and the sensor temperature, correspondingly. Then, the box lid is taken off to release the condensed heat. This temperature cycle results in ∼300 measurements per MUT, and the measured $f_{res}$ distributions are shown in the right axis of Figure 45c. The whole scenario is repeated with similarly concentrated samples of acetone in water, as shown in Figure 45d. Therefore, 5 concentrations of each material constitute 10 different classes when the methanol-in-water and acetone-in-water datasets are combined as the input. The choice of the classifier depends on the data dimension as well as prior information. In this complex multiclass classification problem, however, several algorithms are empirically compared to choose the best-performing model. The proposed artificial neural network employs several different classifiers including multilayer perceptron (MLP), decision tree (DT), K-nearest neighbors (KNN), support vector machine (SVM), random forest and linear discriminant analysis (LDA) to map the received input (5001 measured $|S_{21}|$ values) into a single class (0, 1, 2, …, 9). It is shown in Figure 46a that a high-decision accuracy can be obtained with a wide range of models, which depicts the feasibility of the proposed method in deciphering an adversely affected sensor response.

The confusion matrix involving the whole dataset classification with classes 0→4 for methanol-in-water and 5→9 for acetone is shown in Figure 46b with the number of predictions per label given in each cell. In another attempt, the concentration of mixture is reduced to evaluate the model performance at smaller deviations in the $f_{res}$. This time, low concentrations ranging from 0%→5% with 1% increment are prepared for both methanol and acetone in water. Similar classification is conducted and the results from each model are compared with red color in Figure 46a. This experiment demonstrates that some algorithms still preserve their high performance level such as MLP and SVM even though the variations in the materials are more intermingled. This work demonstrates that machine learning-based algorithms are potent in helping microwave planar devices remove their imperfections due to their planar design.

## 5. Comparative Analysis

In this section, a brief overview of all proposed methodologies and techniques are given that can be used to enhance the performance of microwave sensors. In most cases, these techniques can be easily applied to non-planar sensors with cavity designs as well [36,41,49,115]. Three categories are discussed including sensitivity, resolution and robustness—all of which contribute to the key performance aspects of the sensor. The suggested approach differs depending on the sensor port topology (1-port/2-port), planar/non-planar, the principle of operation and sensing mechanism (frequency/phase/amplitude measurement). Table 4 elaborates on all possibilities to improve the sensor performance with a detailed application and sensing technique.

## 6. Conclusions

In conclusion, several unique and recent approaches have been reviewed that significantly contribute to the functionality and performance of conventional microwave planar sensors. Many of the proposed techniques can also be applied to non-planar sensors working in a microwave regime. The main purpose of the proposed work is discussing specific strategies in three categories including the sensitivity, resolution and robustness enhancement of the sensors. The prototype examples of each method were developed. In particular, increasing the sensitivity of the sensor was elaborated with various methods such as a sensor exploiting coupled resonators that are mostly used for planar two-port sensing. Embedding channels inside the substrate can be used for both planar/non-planar systems to exploit the main capacitive region of the sensor, while being more immune to environmental impacts. In cases where the MUT is infinitesimal, e.g., proteins, gases, etc., analytes are immobilized and physically adsorbed to increase the cumulative impact on the sensor. The resonator pattern, in addition to canonical demonstrations, can be optimized for the highest field concentration and sensitivity. While frequency/amplitude measurements are prone to noise/error, using the phase variation is another method to achieve a highly sensitive sensor. The incorporation of coupled line sections can be used for wide-band spectroscopy. Each method introduces a unique example to enhance the sensitivity of a conventional sensor up to a limited level using either passive or active methods. In contrast to the passive methods, in a nonlinear sensor using intermodulation products, one can arbitrarily increase the sensor sensitivity at the cost of power consumption. In this work, an example with 4-fold sensitivity enhancement was shown to operate as stable as the fundamental frequencies. This aspect of integrating electronics within passive sensors is demonstrated in the next section that explains the performance enhancement with respect to the resolution of sensor response. This section mainly discusses the use of active electronics in order to retrieve the lost power in the resonator. This way, a typical resonator quality factor can increase 20-fold with the use of a proper compensation technique for both transmissive and reflective schemes, using which the sensor resolution increases proportionally. Moreover, oscillators as sensors are also covered, and their time-based stability is boosted with an additional loss-compensating circuit for resonator Q-factor enhancement. The cross-mode operation in two identical transmissive sensor yields high resolution in the readout. Lastly, the recent surge in machine learning has been found to be advantageous in boosting the performance of microwave sensing in terms of leveraging pre-trained patterns towards removing environmental impacts from sensor response, thereby resulting in a robust sensor. Two main techniques including the fuzzy neural network and artificial neural network are discussed that both help resolve environmental impacts such as minor MUT displacement or temperature. With all these characteristics, the reviewed sensor techniques can be applied to various scenarios including medical diagnosis, industrial processing and monitoring, among others.

## Figures and Tables

**Table 1:** Geometry and substrate parameters for the considered DB-DGS-based sensors. Reprinted with permission from Ref. [59].

Sensor Type	$f_{0,\mathrm{air}}$ [GHz]	$W_{s}$ [mm]	$\varepsilon_{r}$	*h* [mm]	*S* [mm]	*l* [mm]	$W_{a}$ [mm]	$l_{a}$ [mm]
A	3.204	3.91	2.20	1.270	0.300	21	3.7	3.7
B	3.226	0.562	3.55	0.254	0.300	20	3.5	3.5
C	3.22	1.13	3.55	0.508	0.600	21.6	3.5	3.5
D	3.24	0.368	6.15	0.254	0.200	20	2.3	2.3
E	3.28	1.184	10.2	1.270	0.200	13.5	1.7	1.7
F	3.29	1.184	10.2	1.270	0.300	15	1.6	1.6

**Table 2:** Comparison of various frequency-variation sensors in terms of sensitivity.

$f_{0,b}$ (GHz)	$|S_{\mathrm{av}}|$ (MHz)	$|{\bar{S}}_{\mathrm{av}}|$ (%)	Dynamic Range	Reference
3.75	38.21	1.019	1.0–78.2	[58]
1.9	1.53	0.081	9.0–80.0	[64]
2.0	4.76	0.238	9.0–79.5	[65]
3.5	9.16	0.261	6.5–80.0	[28]
0.87	0.79	0.091	27.8–80.8	[66]

**Table 3:** Sensor response analysis between external and embedded channels, Reprinted with permission from Ref. [36].

MUT	External		Embedded		Improvement
	$f_{\mathrm{res}}$ (GHz)	S (MHz)	$f_{\mathrm{res}}$ (GHz)	S (MHz)	in S (%)
Bare Sensor	5.2854	-	5.2096	-	-
IPA	5.2798	0.33	5.1895	1.19	259
Ethanol	5.2725	0.56	5.1861	1.02	82
Methanol	5.2644	0.72	5.1848	0.85	18
Acetone	5.2626	1.2	5.1786	1.63	36

**Table 4:** Comparative analysis between various enhancing techniques for microwave planar sensors.

Feature	Proposed Method	Comment	Application	References
Sensitivity Enhancement	Coupled Resonator	Coupling between resonators is used Resonance is only due to coupling	Biomedical	[33,34,67]
	Substrate Embedding	Fluids between resonator and ground MUT disturbs the resonator capacitance	MUT characterization	[36]
	Analyte Immobilization	Intermediate layer to hold MUT Mostly For gases/minuscule analytes	Biomedical Temperature Humidity	[72]
	Sensor Topology Optimization	Sensor hot spot is optimized High concentration of E-fields are generated with optimized geometries	Biomedical	[37]
	Phase Variation	For reflection-based (1-port) sensors Alternating high-low impedance TLs	MUT characterization	[96]
	Coupled-Line Coupler	Enables wide-band spectroscopy Affecting coupling/isolation of coupler	MUT characterization	[110,111]
	Intermodulation Product	Enables arbitrarily enhanced sensitivity Requires active circuit design Sensitivity enhanced, noise maintained	Oil-sand Biomedical	[41]
Resolution Enhancement	Active Transmission Sensor	For planar/non-planar designs Loss-compensation for high resolution Generating ultra-high Q-factors	Biomedical Oil–sand	[46,113,114]
	Active Reflective Sensor	Applicable for single-port devices Leads to ultra-sharp resonance	Ultra lossy MUT	[115]
	Active Tank-Based Oscillator	Improves the Q-factor of oscillators Enhances the sensor jitter	Lossy MUT Characterization	[43]
	Cross-Mode Transmission	Differentiation of *S*_21_ of two sensors For highly lossy MUT with negligible frequency variation	Biomedical	[117]
Robustness Enhancement	Fuzzy Neural Network	Sensor response stabilized with respect to the MUT-location variation The sensor is trained to be sensitive to MUT, rather than location	Industrial application MUT characterization	[36]
	Artificial Neural Network	Material properties vary by temperature Temperature compensation is achieved by ANN on sensor response	Industrial application Biomedical Agriculture	[49]

## Data Availability

Not applicable.
